# TRAF6-mediated ubiquitination of AKT in the nucleus is a critical event underlying the desensitization of G protein-coupled receptors

**DOI:** 10.1186/s12964-024-01592-z

**Published:** 2024-04-02

**Authors:** Chengyan Wu, Li Hu, Bing Liu, Xingyue Zeng, Haixiang Ma, Yongkai Cao, Huijun Li, Xiaohan Zhang

**Affiliations:** 1 School of Pharmaceutical Sciences, Guizhou University, Guiyang, 550025 China; 2 Institute of Translational Medicine, The First Affiliated Hospital of Shenzhen University, Shenzhen, 518035 China; 3 Department of Pharmaceuticals, People’s Hospital of Zunyi City Bo Zhou District, Zunyi, 563000 China

**Keywords:** AKT ubiquitination, Desensitization, GPCRs, TRAF6, Src

## Abstract

**Background:**

Desensitization of G protein–coupled receptors (GPCRs) refers to the attenuation of receptor responsiveness by prolonged or intermittent exposure to agonists. The binding of β-arrestin to the cytoplasmic cavity of the phosphorylated receptor, which competes with the G protein, has been widely accepted as an extensive model for explaining GPCRs desensitization. However, studies on various GPCRs, including dopamine D_2_-like receptors (D_2_R, D_3_R, D_4_R), have suggested the existence of other desensitization mechanisms. The present study employed D_2_R/D_3_R variants with different desensitization properties and utilized loss-of-function approaches to uncover the mechanisms underlying GPCRs homologous desensitization, focusing on the signaling cascade that regulates the ubiquitination of AKT.

**Results:**

AKT undergoes K8/14 ubiquitination by TRAF6, which occurs in the nucleus and promotes its membrane recruitment, phosphorylation and activation under receptor desensitization conditions. The nuclear entry of TRAF6 relies on the presence of the importin complex. Src regulates the nuclear entry of TRAF6 by mediating the interaction between TRAF6 and importin β1. Ubiquitinated AKT translocates to the plasma membrane where it associates with Mdm2 to phosphorylate it at the S166 and S186 residues. Thereafter, phosphorylated Mdm2 is recruited to the nucleus, resulting in the deubiquitination of β-Arr2. The deubiquitinated β-Arr2 then forms a complex with Gβγ, which serves as a biomarker for GPCRs desensitization. Like in D_3_R, ubiquitination of AKT is also involved in the desensitization of β_2_ adrenoceptors.

**Conclusion:**

Our study proposed that the property of a receptor that causes a change in the subcellular localization of TRAF6 from the cytoplasm to the nucleus to mediate AKT ubiquitination could initiate the desensitization of GPCRs.

**Supplementary Information:**

The online version contains supplementary material available at 10.1186/s12964-024-01592-z.

## Introduction

GPCRs play a crucial role in transmitting numerous extracellular signals intracellularly to appropriate downstream effectors and regulating various physiological processes. Excessive stimulation of GPCRs signaling can lead to cellular toxicity or uncontrolled cellular growth. Accordingly, receptor desensitization, which refers to the rapid reduction of receptor responsiveness by repeated stimulation of a GPCRs with its agonist over minutes, has emerged as a protective measure for limiting GPCRs signaling [1, 2]. Traditionally, desensitization can be categorized as either homologous or heterologous. Homologous desensitization is characterized by a diminished response of the receptor exclusively to continuous or repeated exposure to an agonist, which is believed to involve adaptive changes at the level of the receptor itself. On the other hand, heterologous desensitization refers to processes whereby the loss of receptor responsiveness occurs in the absence of agonist occupation of the receptors, typically involving changes in signaling components downstream of the GPCRs. In this study, we focused on examining the mechanisms underlying homologous desensitization of GPCRs.

The classical model to explain homologous desensitization of GPCRs relies primarily on the study of the β_2_ adrenergic receptor (β_2_AR). According to this model, when a receptor is occupied by an agonist, specific serine and threonine residues within its intracellular domains can be phosphorylated by members of the GRKs family. This phosphorylation enhances the interaction between the receptor and β-arrestin. Consequently, the binding of β-arrestin to the transmembrane core of the phosphorylated receptor inhibits further binding to G proteins, leading to desensitization of the response. Receptor phosphorylation and β-arrestin binding are considered the two critical cellular events responsible for GPCRs desensitization. However, G protein-mediated signaling requires the formation of a trimeric complex comprising a single GPCR, β-arrestin, and G protein [3], indicating that β-arrestin and G protein are capable of even broader functions than previously thought. Moreover, some studies have revealed that the classical model cannot be employed to properly explain the desensitization of most GPCRs [4]. For example, GPCRs desensitization occurs independently of receptor phosphorylation through the RGS homology (RH) domain of GRK2/3, which sequesters activated Gaq/11 [5]. Additionally, the attenuation of mGluR5 responses in striatal neurons occurs in a phosphorylation-independent manner [6].

Dopamine, a pivotal neurotransmitter, exerts its effects through the dopamine receptors (D_1_R–D_5_R). Within this family, dopamine D_2_-like receptors, specifically D_2_R, D_3_R, and D_4_R, share similar signaling pathways but exhibit distinct regulatory features. For example, D_2_R undergoes GRK2/3-mediated receptor phosphorylation and promotes β-arrestin recruitment to the plasma membrane [7]. D_4_R, while also facilitating β-arrestin translocation, does so with reduced efficacy compared to D_2_R [8]. Conversely, D_3_R, which inhibits adenylyl cyclase activity and reduces cAMP levels through coupling to the inhibitory G protein subunits Gi and Go, is reported to rarely undergo agonist-induced phosphorylation and β-arrestin recruitment [7]. Nonetheless, D_3_R undergoes DA or other agonists induced instantaneous desensitization through measuring cellular cAMP and MAPK signaling. This suggests that D_3_R may employ distinct desensitization mechanisms that are independent of the classical phosphorylation-dependent and GRK2/β-arrestin2-mediated pathways.

It has been reported that both Gβγ and β-arrestin can mediate a process known as pharmacological sequestration, defined as the movement of cell surface receptors into a more hydrophobic fraction within the plasma membrane, which may predict the homologous desensitization of D_3_R [9]. Furthermore, recent research has uncovered specific cellular components and events implicated in the development of D_3_R homologous desensitization. For example, a tightly bound complex formed by deubiquitinated β-Arr2 and Gβγ that translocates to the nucleus through an importin-dependent mechanism serves as a novel route to sequester Gβγ, disrupt D_3_R signaling, and induce homologous desensitization [10]; the cytoplasm-to-nucleus translocation of Mdm2, leading to the deubiquitination of β-arrestins, could serve as a biomarker to predict D_3_R desensitization [11]. Furthermore, D_3_R has been demonstrated to undergo PKC-mediated heterologous desensitization [12].

In this study, we further uncovered the molecular mechanisms underlying GPCRs homologous desensitization. To verify the credibility of our identified biomarkers for explaining GPCRs desensitization, we altered the desensitization properties of the receptors and assessed whether the biomarkers exhibited corresponding changes. Our findings suggest a close correlation between receptor desensitization and TRAF6-mediated AKT ubiquitination.

## Materials and methods

### Reagents

Rabbit antibodies against HA (H6908), rabbit antibodies against green fluorescent protein (GFP) (G1544), rabbit anti-FLAG M2 antibodies (F7425), agarose beads coated with monoclonal antibodies against the FLAG epitope and dopamine (DA) were purchased from Sigma-Aldrich Chemical Co. (St. Louis, MO, USA). Isoproterenol (II0200) was purchased from Beijing Solarbio Science & Technology Co. (Tongzhou, Beijing, China). LMB (L102387) and PP2 (P125361) were purchased from Aladdin Chemical Reagent Co., Ltd. (Shanghai, China). Quinpirole (85798–08-9) was purchased from Tocris Bioscience. (Bristol, United Kingdom). Triciribine (35943–35-2) was purchased from Rhawn Chemical Reagent Co., Ltd. (Shanghai, China). Rabbit antibodies against Mdm2 (sc-965), mouse antibodies against Lamin B1 (sc374015) and TRAF6 (sc8409) were purchased from Santa Cruz Biotechnology, Inc. (Dallas, TX, USA). Rabbit antibodies against phospho-AKT(S473) (4060S) and phospho-AKT(T308) (9275S) were purchased from Cell Signaling Technology (Danvers, MA, USA). Rabbit antibodies against β-actin (66009–1-LG) were purchased from Proteintech Group, Inc. (Wuhan, China). Rabbit antibodies against phospho-Mdm2 (S166) (AF-3376) were purchased from Affinity Biosciences (Jiangsu, China). Anti-rabbit horseradish peroxidase (HRP)-conjugated secondary antibodies (#65–6120) were obtained from Thermo Fisher Scientific (Waltham, MA, USA) and anti-mouse HRP-conjugated secondary antibodies (115–035-003) were purchased from Jackson ImmunoResearch Laboratories, Inc. (West Grove, PA, USA). Alexa Fluor 555-conjugated anti-rabbit antibodies were purchased from Molecular Probes (Invitrogen, Carlsbad, CA, USA).

### Cell culture

Human embryonic kidney 293 (HEK293) cells were obtained from the National Collection of Authenticated Cell Cultures (Xuhui, Shanghai, China) and cultured in Dulbecco’s modified Eagle medium supplemented 10% fetal bovine serum, 100 U/mL penicillin, and 100 μg/mL streptomycin (Thermo Fisher Scientific). Transfections were conducted using polyethylenimine (Polyscience, Warrington, PA, USA). TRAF6-KD cells and importin β1-KD cells were generated by stably expressing small hairpin RNAs (shRNAs) in PLKO.1 (Sigma-Aldrich Co.) targeting each gene under puromycin selection. CTRL-shRNA cells were prepared by stably transfecting scrambled shRNAs into the corresponding vectors. Only HEK293 cells with passage numbers ranging from 35 to 45 were utilized throughout this study.

### Plasmid constructs

The mammalian expression constructs for D_2_R, D_3_R, β_2_AR and HA-Ub were constructed as described in a previous study [13]. TRAF6 and C70A-TRAF6 (FLAG-tagged) were purchased from Addgene (Beijing Zhongyuan, Ltd., Beijing, China). GFP-TRAF6 constructs, Mdm2 constructs (FLAG, GFP-tagged), AKT constructs (FLAG, GFP, HA-tagged), K8/14R-AKT (FALG-tagged), C147K-D_3_R and K149C-D_2_R were constructed by polymerase chain reaction (PCR) (TL988/TianLong; China). TRAF6-shRNA was purchased from Santa Cruz Biotechnology, Inc. (Dallas, TX, USA), and importin β1-shRNA was obtained from Sigma Aldrich Chemical Co.

### Luciferase reporter gene assay

To indirectly measure cellular cAMP levels, a reporter plasmid containing the firefly luciferase gene under the control of multiple cAMP-responsive elements (CREs) and a control vector, pRL-TK, were used. This method has been previously utilized to determine dopamine receptor signaling pathways, including D_1_R, D_2_R, and D_3_R, producing similar results to a direct assay in which the accumulation of [^3^H]-cAMP was determined through sequential chromatography [14]. The obtained data were normalized and are expressed as a percentage of the forskolin-stimulated cAMP level for each experiment. Dose-response curves were generated using Graph Pad Prism software (Graph Pad Software; San Diego, CA, USA).

### Determination of receptor desensitization

Desensitization was conducted using a previously established method [9]. Specifically, HEK293 cells expressing the corresponding receptor were treated with the agonist for 5 min (initial treatment) to trigger desensitization, followed by washing three times with warm serum-free medium at 37 °C for 5 min each time to ensure the complete removal of agonist that bound to the receptors. Subsequently, the cells were retreated with the agonist for 5 min to provoke a secondary response. The extent of desensitization was determined by comparing the amplitudes of the secondary response in cells pretreated with either vehicle or agonist. “w/+” refers to DA/Quin-treated, washed, and retreated.

### Detection of protein ubiquitination

HA-ubiquitin (HA-Ub) and FLAG-tagged proteins were cotransfected into HEK293 cells expressing the corresponding receptors. Ubiquitination of proteins was determined as described previously [15]. Briefly, cell lysates were solubilized in RIPA buffer supplemented with 1 mM sodium orthovanadate, 1 mM sodium fluoride, 10 mM N-ethylmaleimide, 2 mM phenylmethylsulfonyl fluoride, and 5 μg/mL each of aprotinin and leupeptin. The lysates were then immunoprecipitated with FLAG beads, after which the eluents were analyzed by immunoblotting.

### Immunoprecipitation

HEK293 cells expressing FLAG-tagged protein and corresponding plasmids were lysed (4 °C, 1 h, rotating) with lysis buffer (20 mM HEPES, 150 mM NaCl, 2 mM EDTA, 10% glycerol, 0.5% NonidetP-40, 5 μg/ml aprotinin, 5 μg/ml leupeptin, 20 μg/ml phenylmethylsulfonylfluoride, 10 mM NaF, and 1 mM sodium orthovanadate), after which the cell lysates were incubated with agarose beads coated with FLAG antibodies for 2–3 h at 4 °C. Then, the beads were washed three times with ice-cold washing buffer (50 mM Tris, pH 7.4; 137 mM NaCl; 10% glycerol; and 1% NP-40) for 5 min each. Immunoprecipitates (IPs) and cell lysates were analyzed via sodium dodecyl sulfate-polyacrylamide gel electrophoresis (SDS-PAGE) and subsequently transferred to nitrocellulose membranes (Sigma-Aldrich Chemical Co.), which were incubated with primary antibodies specific for the target proteins at 4 °C for overnight, followed by incubation with HRP-conjugated secondary antibodies. Finally, the target proteins were visualized by a chemiluminescent substrate (Thermo Fisher Scientific). Immunoblots were quantified by densitometry using ImageJ (National Institutes of Health, USA).

### Immunocytochemistry

HEK293 cells were transfected with the corresponding plasmids and cultured on glass coverslips. After 24 ~ 36 h, the cells were fixed with 4% paraformaldehyde in phosphate-buffered saline (PBS) for 20 min at room temperature and permeabilized with 0.1% Triton X-100 in PBS for 90 seconds at room temperature. Then, the cells were blocked with PBS containing 3% FBS and 1% BSA for 1 h and incubated with the corresponding antibodies (1:1000) for 2 h at room temperature. After washing 3 times with PBS, the cells were incubated with Alexa 594-conjugated secondary antibodies (1:500) and visualized by laser-scanning confocal microscopy (TCS SP5/AOBS/Tandem; Germany). The images were analyzed by ImageJ (National Institutes of Health, Bethesda, MA, USA).

### Subcellular fractionation

Subcellular fractionation was conducted as described previously [16]. In Detail, the cells were incubated with buffer-1 (10 mM HEPES/KOH pH 7.8, 1.5 mM MgCl_2_, 10 mM KCl, 0.5 mM dithiothreitol, 0.2 mM phenylmethylsulfonyl fluoride, and 1 mM Na_3_VO_4_) for 20 min and then centrifuged at 2000×g for 5 min. The supernatants were subsequently centrifuged for an additional 10 min at 15,000×g, after which the proteins were saved as the cytoplasmic fraction. The pellet from the initial centrifugation was washed for 15 min with buffer-1 and centrifuged at 15,000×g for 10 min. The pellet was incubated with buffer-2 (20 mM HEPES/KOH pH 7.8, 1.5 mM MgCl_2_, 420 mM NaCl, 0.2 mM EDTA, 25% glycerol, 0.5 mM dithiothreitol, 0.2 mM phenylmethylsulfonyl fluoride, and 1 mM Na_3_VO_4_) for 20 min and then centrifuged. After centrifugation at 24,000×g for 10 min, the supernatant was collected as the nuclear fraction. The reference proteins for the cytosolic and nuclear fractions were β-actin and lamin B1, respectively.

## Statistical analysis

The statistical analysis was performed utilizing independent values corresponding to information obtained from various immunoblots or assays. To control for unwanted sources of variation, the normalized immunoblot values were averaged and are expressed as the fold change relative to the mean value of the control group as 1. The Y-axis label in the figures indicates the “fold mean of the controls”. All the data are expressed as the mean ± SD. Statistical significance was determined using paired two-tailed Student’s t tests for two groups or one-way ANOVA with Tukey’s post hoc test for multiple groups. In all the analyses, only one threshold for statistical significance (*p* < 0.05) was used. GraphPad Prism 8 software (GraphPad Software Inc., San Diego, CA, USA) was used for the analysis.

## Results

### Homologous desensitization of D_3_R accompanies TRAF6-mediated AKT ubiquitination and phosphorylation at T308/S473

**Fig. 1:**
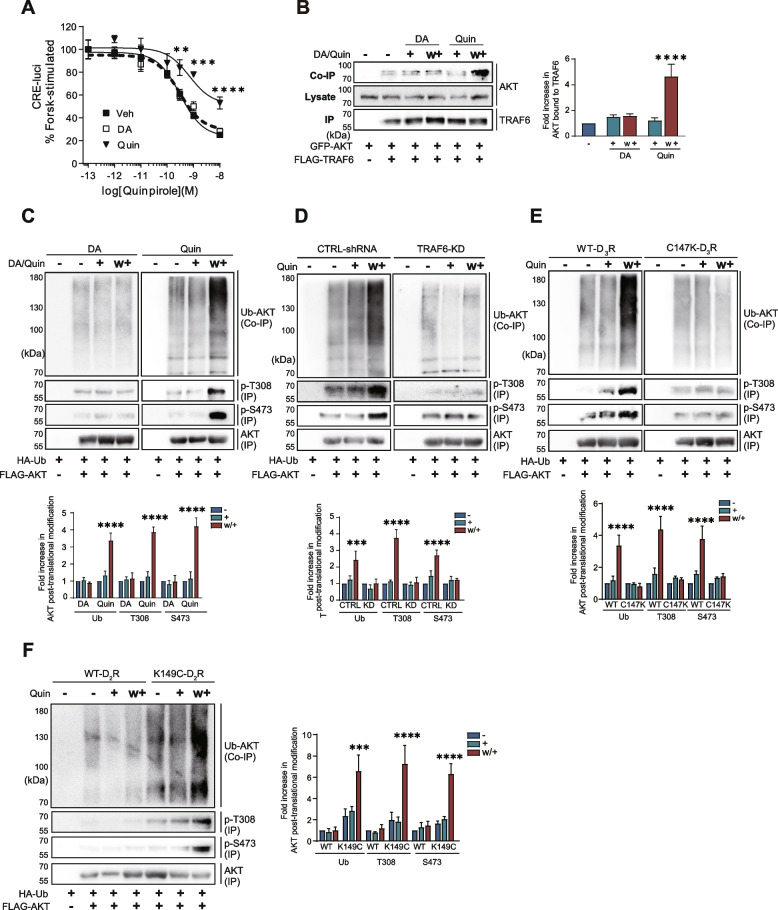
Characterization of TRAF6-mediated AKT ubiquitination and phosphorylation under receptor desensitization. **A** HEK293 cells expressing D_3_R (1.9 ~ 2.1 pmol/mg protein) were treated with 100 nM Quin or 30 nM DA for 5 min, washed three times with warm serum-free medium, and then treated with increasing concentrations of Quin. The cellular levels of cAMP were indirectly evaluated by using the CRE-luciferase reporter gene assay. Quin treatment group was significantly different from the Veh group at concentrations of quinpirole between 10^−10^ and 10^−8^ M (**p < 0.01, ***p < 0.001, ****p < 0.0001 (*n* = 3)). **B** HEK293 cells expressing D_3_R (1.9 ~ 2.1 pmol/mg protein) were transfected with GFP-AKT and FLAG-TRAF6, then the cells were treated with 30 nM DA or 100 nM Quin for 5 min according to desensitization protocol. Cell lysates were immunoprecipitated with anti-FLAG beads. Co-IP/lysate and IP were immunoblotted with antibodies against GFP or FLAG, respectively. ****p < 0.0001 compared to the vehicle treated group (*n* = 3). (C-F) Cell lysates were immunoprecipitated with anti-FLAG beads. Co-IP was immunoblotted with antibodies against HA. IPs were immunoblotted with antibodies against p-AKT(T308), p-AKT(S473) and FLAG, respectively. “+” and “w/+” refer to DA/Quin-treated and washed/retreated cells. **C** HEK293 cells expressing D_3_R (1.9 ~ 2.1 pmol/mg protein) were transfected with HA-Ub and FLAG-AKT. The cells were treated with 30 nM DA or 100 nM Quin for 5 min according to desensitization protocol. ****p < 0.0001 compared to the corresponding vehicle treated group (*n* = 3). **D** CTRL-shRNA and TRAF6-KD cells were co-transfected with D_3_R, HA-Ub, and FLAG-AKT. The cells were treated with 100 nM Quin for 5 min according to desensitization protocol. ***p < 0.001, ****p < 0.0001 compared to the corresponding vehicle treated group (*n* = 3). **E** HEK293 cells were co-transfected with WT-D_3_R or C147K-D_3_R, HA-Ub, and FLAG-AKT. ****p < 0.0001 compared to the corresponding vehicle treated group (*n* = 3). **F** HEK293 cells were co-transfected with WT-D_2_R or K149C-D_2_R, HA-Ub, and FLAG-AKT. ***p < 0.001, ****p < 0.0001 compared to the corresponding vehicle treated group (*n* = 3)

The serine and threonine protein kinase AKT, also known as protein kinase B (PKB), has been shown to play a crucial role in the control of dopamine D_3_ receptor (D_3_R) homologous desensitization [17]. However, the molecular details involved in this process have not yet been characterized. To better understand the regulatory effects of AKT on D_3_R homologous desensitization, we first investigated the relationship between AKT ubiquitination and receptor desensitization. D_3_R desensitization occurred in response to pretreatment with 100 nM quinpirole (Quin) rather than 30 nM dopamine (DA) (Fig. 1A) [10, 11]. Subsequent experiments were conducted by pretreating cells with 100 nM quinpirole to assess the desensitization of D_3_R and D_2_R variant. As shown in Fig. 1B, the E3 ubiquitin ligase TRAF6 interacted with AKT under D_3_R desensitization conditions (Quin/w+), resulting in an increase in the ubiquitination of AKT (Fig. 1C, right/upper panels). Moreover, the phosphorylation of AKT at T308 and S473, which serve as markers of its activation [18], also increased under D_3_R desensitization conditions (Fig. 1C, right/lower panels). Knockdown of TRAF6 blocked desensitization-induced AKT ubiquitination and T308/S473 phosphorylation (Fig. 1D right panels). These results suggest that homologous desensitization of D_3_R is correlated with TRAF6-mediated AKT ubiquitination and T308/S473 phosphorylation.

To further evaluate the correlations between AKT ubiquitination and homologous desensitization, other receptors with differing desensitization properties were evaluated. Unlike D_3_R, neither D_2_R nor C147K-D_3_R undergo desensitization [9, 19]. Substituting the C147 residue with lysine may affect the agonist-induced conformational change of the receptor, which is essential for dissociating D_3_R from its signaling pathway, thereby triggering desensitization [20]. As expected, C147K-D_3_R failed to mediate the ubiquitination or phosphorylation of AKT under desensitization conditions (Fig. 1E, right panels). Similarly, activation of D_2_R was unable to mediate the ubiquitination or phosphorylation of AKT (Fig. 1F, left four panels). In contrast, K149C-D_2_R, a mutant of D_2_R that does undergo desensitization [21], was observed to mediate the ubiquitination and phosphorylation of AKT (Fig. 1F, right three panels). All of these results suggest that D_2_-like receptors prone to eliciting desensitization are accompanied by TRAF6-mediated AKT ubiquitination and phosphorylation at T308 and S473, which serve as AKT activation markers.

### TRAF6-mediated AKT K8/14 ubiquitination occurs upstream of AKT phosphorylation

**Fig. 2:**
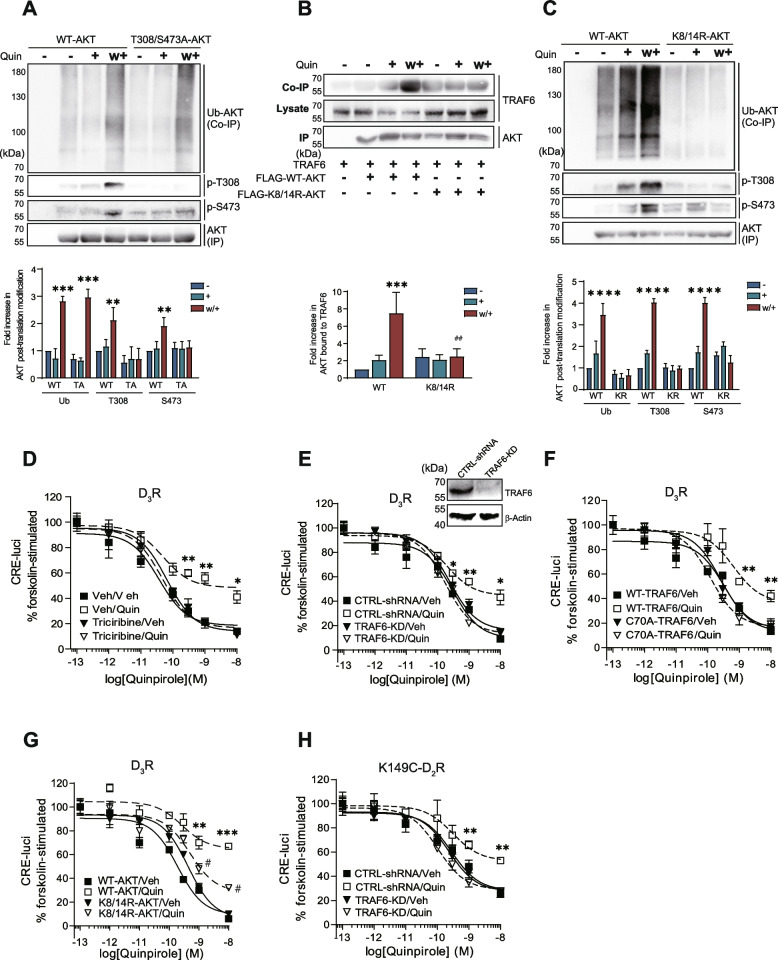
TRAF6-mediated AKT K8/14 ubiquitination leads to AKT phosphorylation, which is required for receptor desensitization. (A-C) Cells were treated with 100 nM Quin for 5 min according to desensitization protocol. Cell lysates were immunoprecipitated with anti-FLAG beads. Co-IP/IP was immunoblotted with corresponding antibodies. **A** HEK293 cells expressing D_3_R (1.9 ~ 2.1 pmol/mg protein) were transfected with HA-Ub and FLAG-WT-AKT or FLAG-T308A/S473A-AKT. Cell lysates were immunoprecipitated with anti-FLAG beads. ***p < 0.001, **p < 0.01 compared to the corresponding “WT/−” group (*n* = 3). **B** HEK293 cells expressing D_3_R (1.9 ~ 2.1 pmol/mg protein) were transfected with TRAF6 and FLAG-WT-AKT or FLAG-K8/14R-AKT. ***p < 0.001 compared to “WT/−” group, ^##^*p* < 0.01 compared to “WT/w+” group (*n* = 3). **C** HEK293 cells expressing D_3_R (1.9 ~ 2.1 pmol/mg protein) were transfected with HA-Ub and FLAG-WT-AKT or FLAG-K8/14R-AKT. ****p < 0.0001 compared to corresponding veh group (*n* = 3). (D-H) After the cells were pretreated, they were treated with 100 nM Quin for 5 min, washed three times with warm serum-free medium, and then treated with increasing concentrations of Quin. The cellular levels of cAMP were indirectly evaluated by using the CRE-luciferase reporter gene assay. ***p < 0.001, **p < 0.01, *p < 0.05 (*n* = 3) (**D**) HEK293 cells expressing D_3_R were pretreated with either vehicle or 200 nM triciribine for 6 h. Veh/Quin group was significantly different from Veh/veh group at treatment concentrations of quinpirole between 3 × 10^−10^ and 10^−8^ M. **E** CTRL-shRNA and TRAF6-KD cells were transfected with D_3_R. CTRL-shRNA/Quin group was significantly different from CTRL-shRNA /Veh group at concentrations ranging from 3 × 10^−10^ to 10^−8^ M. The cell lysates were immunoblotted with antibodies against TRAF6 and β-actin. The knockdown efficiency of TRAF6 was approximately 87%. **F** TRAF6-KD cells were transfected with D_3_R and WT-TRAF6 or C70A-TRAF6. The WT-TRAF6/Quin group was significantly different from WT-TRAF6/Veh group at concentrations of quinpirole between 10^−9^ and 10^−8^ M. **G** HEK293 cells expressing D_3_R were transfected with WT-AKT or K8/14R-AKT. The WT-AKT/Quin group was significantly different from other groups at concentrations of quinpirole between 10^−9^ and 10^−8^ M. **H** CTRL-shRNA and TRAF6-KD cells were transfected with K149C-D_2_R. The CTRL-shRNA/Quin group was significantly different from CTRL-shRNA/veh group at concentrations of quinpirole between 10^−9^ and 10^−8^ M

Since D_3_R and D_2_R variant desensitization is accompanied by AKT ubiquitination and phosphorylation, we next determined the crosstalk between AKT ubiquitination and phosphorylation under receptor desensitization conditions. To test this possibility, two AKT mutations were used. The T308A/S473A-AKT mutant, in which the two phosphorylation sites were mutated to alanine, still exhibited ubiquitination independent of its phosphorylation (Fig. 2A). K8 and K14 residues within the PH domain are responsible for the basal ubiquitination of AKT [22]. Mutation of K8/14 to arginine impaired the receptor desensitization conditions-induced interaction of AKT with TRAF6 (Fig. 2B) and AKT ubiquitination (Fig. 2C). Furthermore, the loss of AKT ubiquitination impaired AKT phosphorylation in response to repeated exposure to the D_3_R agonist (Fig. 2C). All of these data suggest that TRAF6-mediated AKT ubiquitination is an upstream event of AKT phosphorylation under receptor desensitization conditions.

### AKT ubiquitination is critical for promoting D_3_R and D_2_R variant desensitization

To further determine the role of AKT ubiquitination in D_3_R and D_2_R variant desensitization, we first pretreated cells with triciribine, an AKT inhibitor, and found that this pretreatment impaired D_3_R desensitization (Fig. 2D), which is consistent with the findings of previous publications [17]. Furthermore, TRAF6 knockdown strongly inhibited AKT ubiquitination (Fig. 1D) and blocked D_3_R desensitization (Fig. 2E). In TRAF6-KD cells, co-expression of WT-TRAF6, but not C70A-TRAF6 lacking E3 ligase activity, restored D_3_R desensitization (Fig. 2F). K8/14R-AKT, which failed to undergo TRAF6-mediated ubiquitination (Fig. 2C), did not produce a similar potentiation of D_3_R desensitization as that observed with WT-AKT (Fig. 2G). Additionally, K149C-D_2_R failed to undergo desensitization when TRAF6 was knocked down (Fig. 2H). All of these results indicate that TRAF6-mediated AKT ubiquitination contributes to the desensitization of D_3_R and D_2_R variant.

### TRAF6-mediated AKT ubiquitination occurs in the nucleus under desensitization conditions

**Fig. 3:**
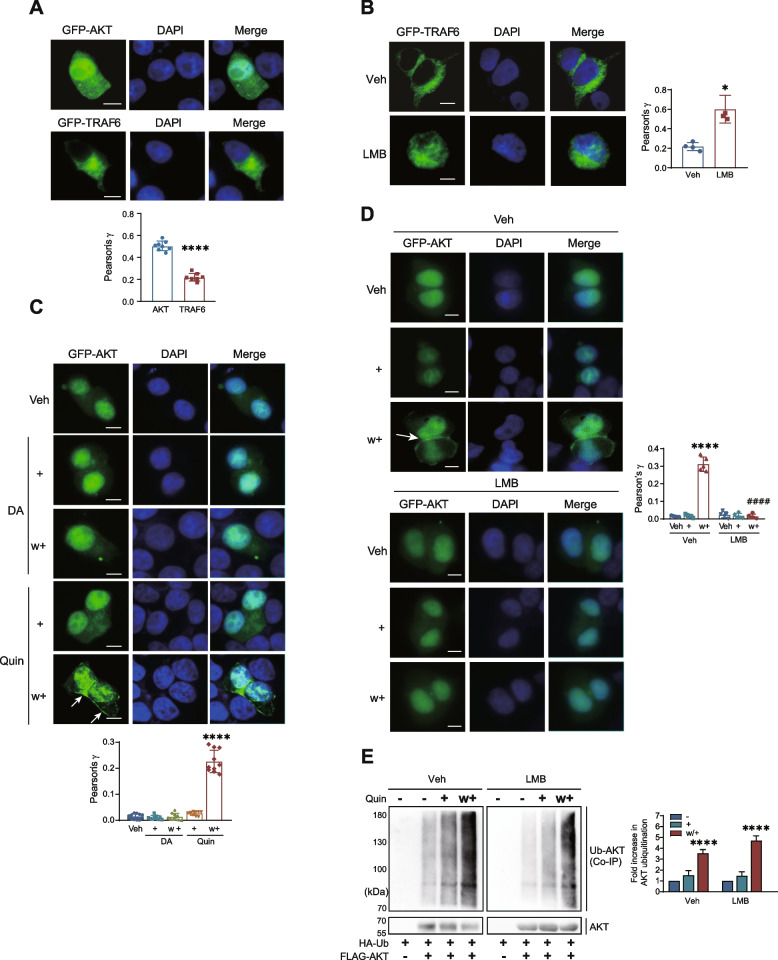
Determination of the subcellular localization of AKT and TRAF6. (A-D) After transfected cells were treated with corresponding drugs, cells were fixed with 4% paraformaldehyde in phosphate-buffered saline (PBS) for 20 min at room temperature. After being washed 3 times with PBS, cells were visualized via a laser-scanning confocal microscope (TCS SP5/AOBS/Tandem; Germany). The scale bar represents 10 μm. “+” and “w/+” refer to drug-treated and washed/retreated cells, respectively. **A** HEK293 cells were transfected with GFP-AKT or GFP-TRAF6. The colocalization of AKT or TRAF6 with DAPI was determined by Pearson correlation coefficient. ****p < 0.0001 compared to AKT transfection group (*n* = 8). **B** HEK293 cells were transfected with GFP-TRAF6. After 24 ~ 36 h, the cells were treated with 30 ng/ml LMB for 3 h. The colocalization of TRAF6 with DAPI was determined by Pearson correlation coefficient. *p < 0.05 compared to vehicle-treated group (*n* = 4). **C** HEK293 cells expressing D_3_R were transfected with GFP-AKT. After 24 ~ 36 h, Cells were treated with 30 nM DA or 100 nM Quin for 5 min, washed, and re-challenged with 30 nM DA or 100 nM Quin for 5 min. ****p < 0.0001 compared to vehicle-treated group (*n* = 10). The membrane localization of AKT was determined by Pearson correlation coefficient. **D** HEK293 cells expressing D_3_R were transfected with GFP-AKT. After 24 ~ 36 h, the cells were pretreated with 30 ng/ml LMB for 3 h, then treated with 100 nM Quin for 5 min, washed, and re-challenged with 100 nM Quin for 5 min. ****p < 0.0001 compared to Veh/veh treated group, ^####^*p* < 0.0001 compared to Veh/w + group (*n* = 5). Membrane localization of AKT was determined by Pearson correlation coefficient. **E** HEK293 cells expressing D_3_R were transfected with HA-Ub and FLAG-AKT. The cells were pretreated with either vehicle or 30 ng/ml LMB for 3 h, followed by treatment with 100 nM Quin for 5 min, washed, and re-challenged. ****p < 0.0001 compared to the corresponding Veh group (*n* = 3)

**Fig. 4:**
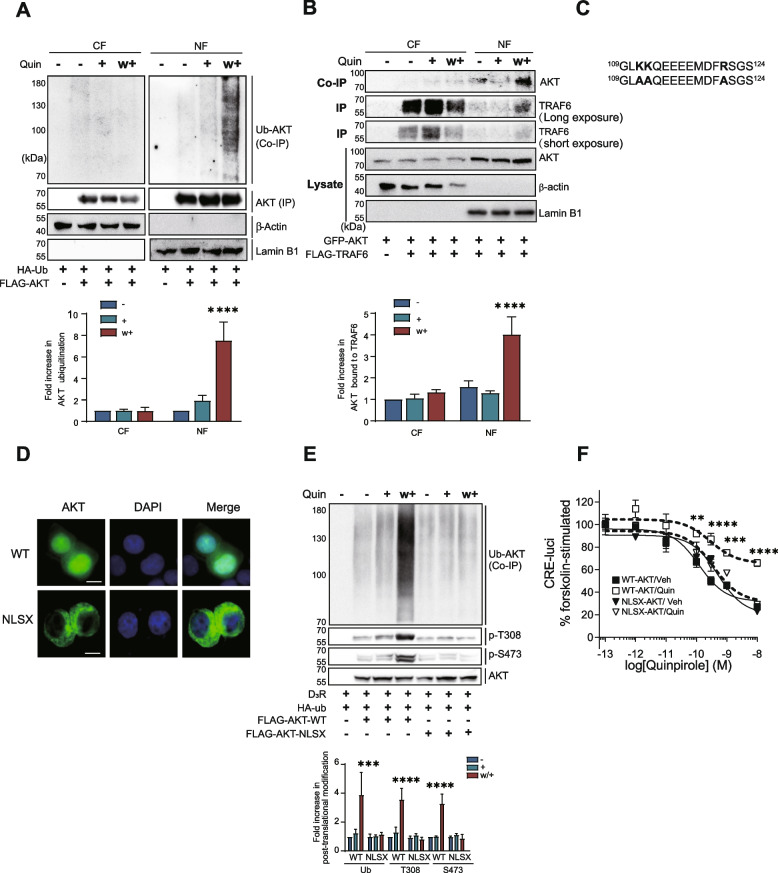
AKT ubiquitination occurs in the nucleus under receptor desensitization conditions. **A** HEK293 cells expressing D_3_R were transfected with HA-Ub and FLAG-AKT. The cells were treated with 100 nM Quin for 5 min, washed, and re-challenged. Cell lysates were fractionated according to the protocol described in the “Materials and methods” section. AKT ubiquitination was assessed using nuclear and cytosolic fractions. NF, nuclear fraction; CF, cytosolic fraction. ****p < 0.0001 compared to “Veh/NF” group (*n* = 3). **B** HEK293 cells expressing D_3_R were transfected with GFP-AKT and FLAG-TRAF6. Cell lysates were fractionated according to the protocol described in the “Materials and methods” section. The interaction of TRAF6 with AKT was assessed in nuclear and cytosolic fractions. NF, nuclear fraction; CF, cytosolic fraction. ****p < 0.0001 compared to corresponding “Veh/NF” group (*n* = 3). **C** The bold letters represent the critical residues in the putative NLS of AKT (upper line). These residues were mutated to alanine residues in NLSX-AKT (lower line). **D** HEK293 cells were transfected with GFP-WT-AKT or GFP-NLSX-AKT. The cells were fixed with 4% paraformaldehyde in phosphate-buffered saline (PBS) for 20 min at room temperature. After being washed 3 times with PBS, the cells were visualized via a laser-scanning confocal microscope (TCS SP5/AOBS/Tandem; Germany). The scale bar represents 10 μm. **E** HEK293 cells expressing D_3_R were transfected with HA-Ub and FLAG-WT-AKT or FLAG-NLSX-AKT. The cells were treated with 100 nM Quin for 5 min, washed, and re-challenged. Cell lysates were immunoprecipitated with anti-FLAG beads. Co-IP/IPs was immunoblotted with corresponding antibodies. ***p < 0.001, ****p < 0.0001 compared to the corresponding Veh group (*n* = 3). **F** HEK293 cells expressing D_3_R were transfected with WT-AKT or NLSX-AKT in AKT-KD cells. The cellular levels of cAMP were indirectly evaluated by using the CRE-luciferase reporter gene assay. WT/Quin group was significantly different from WT/Veh group at concentrations of quinpirole between 10^−10^ and 10^−8^ M.****p < 0.0001, ***p < 0.001, **p < 0.01 (*n* = 3)

In the resting state, AKT is predominantly expressed in the nucleus, whereas TRAF6 is predominantly expressed in the cytoplasm (Fig. 3A) [23, 24]. Leptomycin B (LMB) has been employed to block NES-mediated nuclear export by binding to CRM1, a receptor for the NES [25]. After the cells were treated with 30 ng/mL LMB for 3 h, TRAF6 was observed in both the nucleus and the cytoplasm (Fig. 3B), indicating that TRAF6 shuttles between the nucleus and the cytoplasm. Moreover, AKT shuttles between the nucleus and cytoplasm [26, 27]. As shown in Fig. 3C, AKT was observed in the nuclei of cells expressing D_3_R under non-desensitizing condition (DA/w+). In contrast, AKT was translocated from the nuclear to the cytoplasm and cell membrane under desensitization conditions (Quin/w+). Thus, D_3_R desensitization conditions may induce interactions between TRAF6 and AKT through two pathways, namely, through which AKT exits the nucleus and interacts with TRAF6 in the cytoplasm, or through which TRAF6 enters the nucleus and interacts with AKT in the nucleus under desensitization conditions. To test this possibility, we utilized LMB treatment to block the nuclear export of TRAF6 and AKT, thereby allowing them to be retained in the nucleus (Fig. 3B & D). Under these experimental conditions, AKT ubiquitination still increased with repeated agonist treatment (Fig. 3E). Next, we detected the ubiquitination of AKT and the interaction between TRAF6 and AKT following repeated exposure to the D_3_R agonist using biochemical fractionation and western blot analysis. Repeated exposure to the D_3_R agonist resulted in increased levels of AKT ubiquitination (Fig. 4A) and enhanced interaction of TRAF6 with AKT, specifically in the nuclear fraction rather than the cytoplasmic fraction (Fig. 4B). These results indicate that AKT ubiquitination likely occurs in the nucleus.

NLSX-AKT, an AKT mutant with a mutation in the NLS, contains three amino acid residues (Fig. 4C, amino acid residues 111–112 and 121 were mutated to alanine, bold characters). Unlike WT-AKT, which was observed in the nucleus, NLSX-AKT was exclusively observed in the cytoplasm (Fig. 4D). Next, AKT was retained in the cytoplasm by utilizing NLSX-AKT, and the resulting effects on AKT ubiquitination and D_3_R desensitization were examined. As shown in Fig. 4E, the ubiquitination of NLSX-AKT did not increase with repeated exposure to the D_3_R agonist. WT-AKT, but not NLSX-AKT, restored D_3_R desensitization in AKT-KD cells (Fig. 4F). All of these results suggest that ubiquitination of AKT occurs in the nucleus, which is required for promoting receptor homologous desensitization.

### Nuclear entry of TRAF6 is a critical step in facilitating D_3_R and D_2_R variant desensitization

**Fig. 5:**
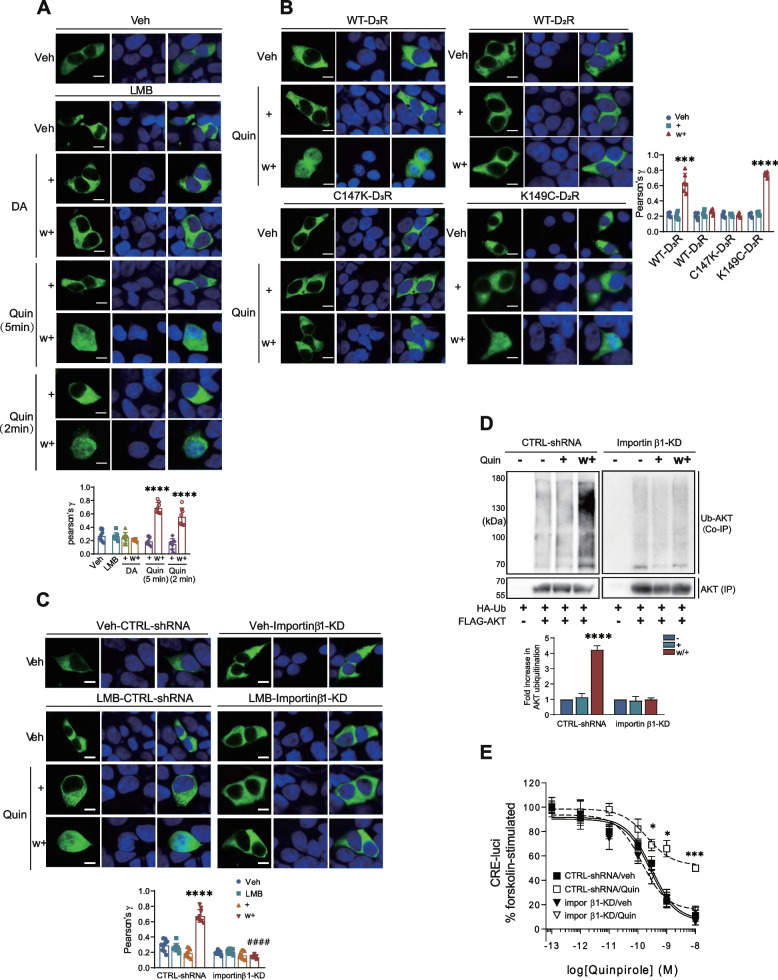
TRAF6 enters the nucleus in an importin β1-dependent manner, which promotes AKT ubiquitination. (A-C) After transfected cells were treated with corresponding drugs, cells were fixed with 4% paraformaldehyde in phosphate-buffered saline (PBS) for 20 min at room temperature. After being washed 3 times with PBS, cells were visualized by laser-scanning confocal microscope (TCS SP5/AOBS/Tandem; Germany). The scale bar represents 10 μm. “+” and “w/+” refer to drug-treated and washed/retreated cells, respectively. **A** HEK293 cells expressing D_3_R were transfected with GFP-TRAF6. The colocalization of TRAF6 with DAPI was determined by Pearson correlation coefficient. ****p < 0.0001 compared to vehicle-treated group (*n* = 8). **B** HEK293 cells were transfected with GFP-TRAF6 and receptors. The colocalization of TRAF6 with DAPI was determined by Pearson correlation coefficient. ***p < 0.001, ****p < 0.0001 compared to corresponding veh group (*n* = 6). **C** CTRL-shRNA and importin β1-KD cells were co-transfected with D_3_R and GFP-TRAF6. The cells were pretreated with either vehicle or 30 ng/ml LMB for 30 min, followed by treatment with 100 nM Quin for 5 min, washed, and re-challenged. The colocalization of TRAF6 with DAPI is shown as Pearson’s coefficient. ****p < 0.0001 compared to “CTRL-shRNA/veh” group, ^####^*p* < 0.0001 compared to “CTRL-shRNA/w+” group (*n* = 9). **D** CTRL-shRNA and importin β1-KD cells were co-transfected with D_3_R, HA-Ub and FLAG-AKT. Cell lysates were immunoprecipitated with anti-FLAG beads. Co-IP was immunoblotted with antibodies against HA. IP was immunoblotted with antibodies against FLAG. ****p < 0.0001 compared to “CTRL-shRNA/−” group (*n* = 3). **E** CTRL-shRNA and importin β1-KD cells were transfected with D_3_R. The cellular levels of cAMP were evaluated by using the CRE-luciferase reporter gene assay. CTRL-shRNA/Quin groups were significantly different from other groups at treatment concentrations of quinpirole between 3 × 10^−10^ and 10^−8^ M. (*p < 0.05, ***p < 0.001, *n* = 3)

If receptor desensitization condition-induced/TRAF6-mediated AKT ubiquitination occurs in the nucleus, the nuclear entry of TRAF6 is expected to be facilitated under desensitization conditions (Quin/w+). To test this possibility, cells were pretreated with LMB for a shorter period (30 min) than after 3 h, followed by treatment with quinpirole. In this setting, the effects of receptor desensitization on the nuclear entry of TRAF6 can be assessed more clearly. As shown in Fig. 5A, TRAF6 was observed in the cytoplasm of cells expressing D_3_R at rest and translocated to the nucleus under desensitization conditions (Quin/w+). As expected, stimulation with non-desensitizing receptors (D_2_R and C147K-D_3_R) did not facilitate the nuclear accumulation of TRAF6 (Fig. 5B, right/upper panels, left/lower panels). In contrast, stimulation with desensitizing receptors (D_3_R and K149C-D_2_R) was observed to facilitate the nuclear localization of TRAF6 (Fig. 5B, left/upper panels, right/lower panels).

The nuclear import of several cytoplasmic proteins, such as β-arrestin2, is generally mediated by the classical nuclear import pathway, which employs importin α and importin β [28, 29]. Next, we utilized importin β1 depleted cells and observed that TRAF6 was located only in the cytoplasm under desensitization conditions (Quin/w+) (Fig. 5C). If nuclear accumulation of TRAF6 is essential for receptor desensitization through the regulation of AKT ubiquitination, both receptor desensitization and ubiquitination of AKT should decrease upon interruption of TRAF6 nuclear accumulation. Upon knockdown of cellular importin β1, AKT ubiquitination (Fig. 5D) and D_3_R desensitization (Fig. 5E) were abolished. These results indicate that the nuclear translocation of TRAF6 is required for promoting D_3_R and D_2_R variant desensitization in an importin β1-dependent manner. The nuclear translocation of this protein could serve as a desensitization marker for D_3_R and D_2_R variant by controlling AKT ubiquitination.

### Src regulates the interaction of TRAF6 with importin β1

**Fig. 6:**
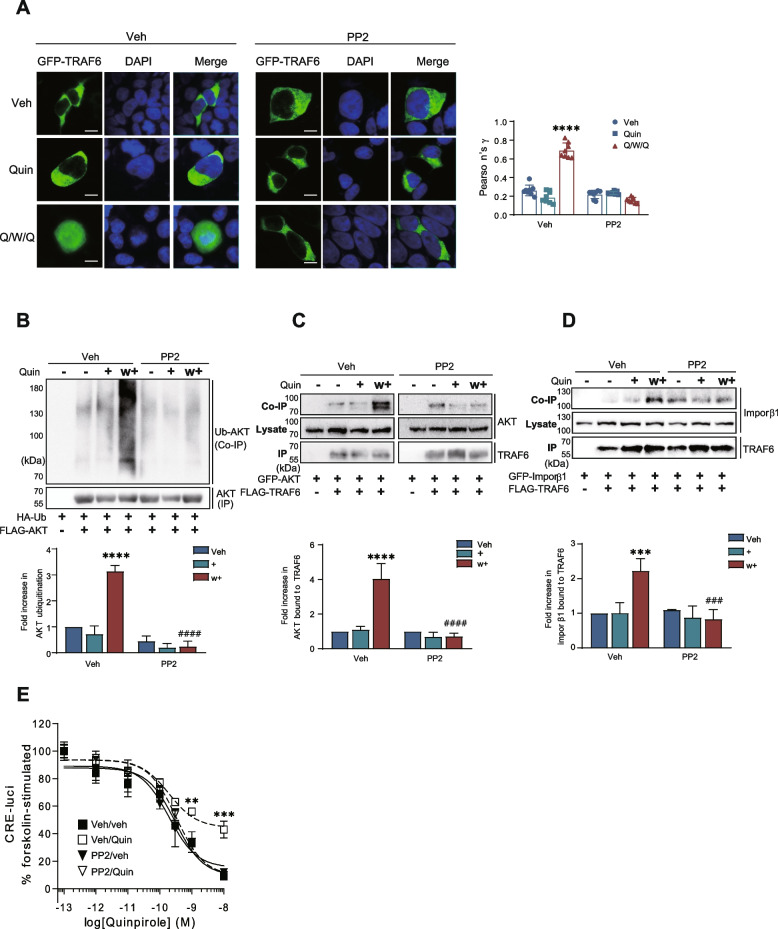
Functional roles of Src in the nuclear import of TRAF6. **A** HEK293 cells expressing D_3_R were transfected with GFP-TRAF6. The cells were treated with 30 ng/ml LMB for 30 min, followed by treatment with either vehicle or 10 μM PP2 for 30 min. Thereafter, the cells were treated with 100 nM Quin for 5 min, washed, and re-challenged. Colocalization between TRAF6 and DAPI is shown as Pearson’s coefficient. ****p < 0.0001 compared to “Veh/veh” group (*n* = 8). The scale bar represents 10 μm. **B** HEK293 cells expressing D_3_R were transfected with HA-Ub and FLAG-AKT. The cells were pretreated with either vehicle or 10 μM PP2 for 30 min, followed by treatment with 100 nM Quin for 5 min, washed, and re-challenged with 100 nM Quin for 5 min. Co-IP and IP were immunoblotted with antibodies against HA and FLAG, respectively. ****p < 0.0001 compared to corresponding Veh/veh group, ^####^*p* < 0.0001 compared to Veh/ w + group (*n* = 3). **C** HEK293 cells expressing D_3_R were transfected with GFP-AKT and FLAG-TRAF6. The cells were pretreated with either vehicle or 10 μM PP2 for 30 min, followed by treatment with Quin, washed, and re-challenged with Quin. Co-IP/Lysate and IP were immunoblotted with antibodies against GFP and FLAG, respectively. ****p < 0.0001 compared to Veh/veh group, ^####^*p* < 0.0001 compared to Veh/ w + group (*n* = 3). **D** HEK293 cells expressing D_3_R were transfected with GFP-importin β1 and FLAG-TRAF6. The cells were pretreated with either vehicle or 10 μM PP2 for 30 min, followed by treatment with Quin, washed, and re-challenged with Quin. Co-IP/Lysate and IP were immunoblotted with antibodies against GFP and FLAG, respectively. ***p < 0.001 compared to Veh/veh group, ^###^*p* < 0.001 compared to Veh/w + group (*n* = 3). **E** HEK293 cells expressing D_3_R were pretreated with vehicle and 10 μM PP2 for 30 min. The cellular levels of cAMP were evaluated by using the CRE-luciferase reporter gene assay. Veh/Quin group was significantly different from other groups at treatment concentrations of Quinpirole between 10^−9^ and 10^−8^ M. (**p < 0.01, ***p < 0.001, *n* = 3)

Previous studies have indicated that Src kinase activity is elevated upon activation of receptors with desensitization properties [17]. Additionally, Src can interact with TRAF6 [30]. We next tested whether Src kinase is responsible for the nuclear entry of TRAF6. As shown in Fig. 6A, pretreatment with the Src inhibitor PP2 blocked the nuclear entry of TRAF6 under desensitization conditions. Furthermore, PP2 pretreatment suppressed AKT ubiquitination and the interaction of TRAF6 with AKT (Fig. 6B & C). These results suggest that Src plays an important role in the regulation of TRAF6-mediated AKT ubiquitination through controlling the nuclear entry of TRAF6.

Since TRAF6 enters the nucleus in an importin β1-dependent manner, we determined the effect of PP2 on the interaction of TRAF6 with importin β1 and found that pretreatment with PP2 significantly disrupted this interaction (Fig. 6D). Additionally, D_3_R desensitization was also blocked by pretreatment with PP2 (Fig. 6E).

### Ubiquitinated AKT moves out of the nucleus to phosphorylate Mdm2, leading to the translocation of the latter from cytosol to nucleus

**Fig. 7:**
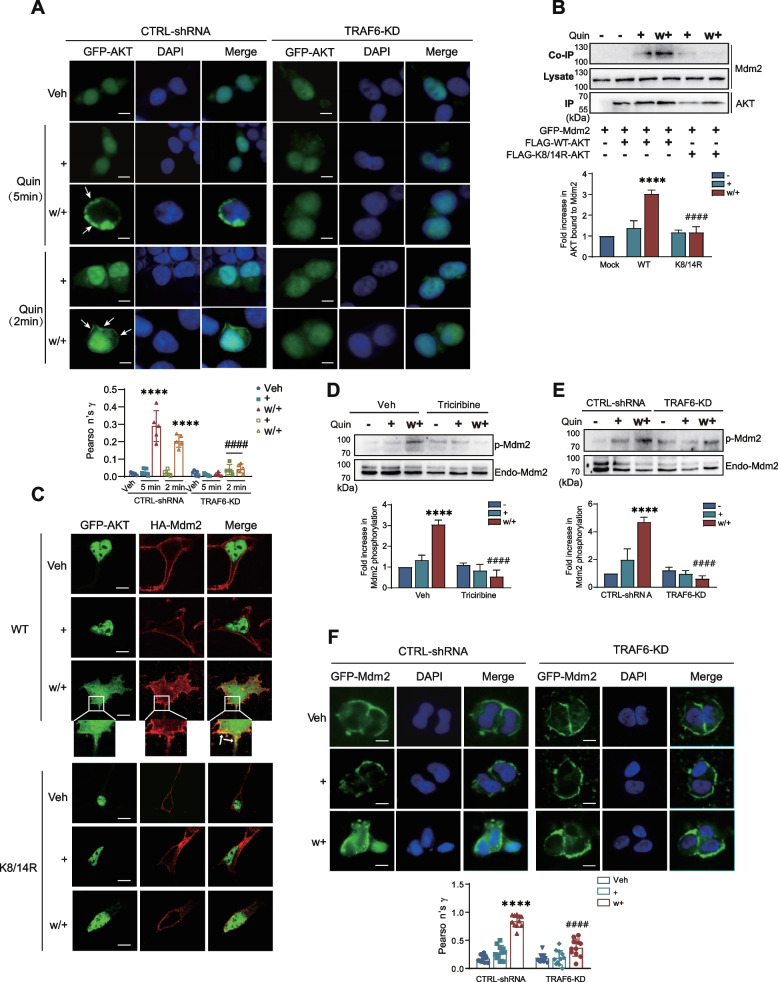
Ubiquitination of AKT is required for its translocation to the cell membrane and subsequent phosphorylation of Mdm2. **A** CTRL-shRNA and TRAF6-KD cells were transfected with D_3_R and GFP-AKT. The cells were treated with 100 nM Quin for 5 min or 2 min, washed, and re-challenged. The scale bar represents 10 μm. The membrane localization of AKT was determined by Pearson correlation coefficient. Data show one representative example out of three independent experiments. ****p < 0.0001 compared to Veh/CTRL-shRNA group, ^####^*p* < 0.0001 compared to w+/ CTRL-shRNA group (*n* = 5). **B** HEK293 cells expressing D_3_R were transfected with GFP-Mdm2 and FLAG-WT-AKT or FLAG-K8/14R-AKT. Then the cells were treated with 100 nM Quin for 5 min, washed, and re-challenged. ****p < 0.0001 compared to Mock group, ^####^*p* < 0.0001 compared to “WT/w+” group (*n* = 3). **C** Cells expressing D_3_R were transfected with GFP-WT-AKT or GFP-K8/14R-AKT and HA-Mdm2. Cells were treated with 100 nM Quin for 2 min, washed, and re-challenged. Cells were labeled with rabbit monoclonal antibodies against HA (1:1000 dilution), followed by incubation with Alexa 594-conjugated anti-rabbit antibodies (1:500 dilution). Arrows in the “w/+” panel represent colocalization of AKT and Mdm2. The scale bar represents 10 μm. **D** HEK293 cells expressing D_3_R were pretreated with 200 nM Triciribine for 6 h, followed by treatment with 100 nM Quin for 5 min, washed, and re-challenged with Quin for 5 min. Cell lysates were immunoblotted with antibodies against phospho-Mdm2 (S166) and Mdm2, respectively. ****p < 0.0001 compared to “Veh/−” group, ^####^*p* < 0.0001 compared to “Veh/w+” group (*n* = 3). **E** CTRL-shRNA and TRAF6-KD cells were transfected with D_3_R. Cells were treated with 100 nM Quin for 5 min washed, and re-challenged. Then cell lysates were immunoblotted with antibodies against phospho-Mdm2 (S166) and Mdm2, respectively. ****p < 0.0001 compared to “CTRL-shRNA/−” group, ^####^*p* < 0.0001 compared to “CTRL-shRNA/w+” group (*n* = 3). **F** CTRL-shRNA and TRAF6-KD cells were co-transfected with D_3_R and GFP-Mdm2. Cells were treated with 100 nM Quin for 2 min, washed, and re-challenged. Co-localization between Mdm2 and DAPI is shown as Pearson’s coefficient. ****p < 0.0001 compared to corresponding “CTRL-shRNA/Veh” group, ^####^*p* < 0.0001 compared to “CTRL-shRNA/w+” group (*n* = 11). The scale bar represents 10 μm

Under desensitization conditions, cytosolic Mdm2 returns to the nucleus, resulting in the deubiquitination of cytosolic β-arrestins, which serves as a regulatory mechanism for GPCRs desensitization [10]. In this process, AKT is the critical kinase that recruits Mdm2 to the nucleus by phosphorylating it at the S166 and S186 residues [31]. We are curious whether the ubiquitination of AKT is required or not; for this, we observed the subcellular localization of AKT in TRAF6-KD cells. As shown in Fig. 7A, under desensitization conditions (Quin/w+), AKT moved out of the nucleus and localized near the cell membrane. When TRAF6 was knocked down, D_3_R desensitization condition-induced nuclear export of AKT was abolished. These data suggest that TRAF6-mediated AKT ubiquitination is responsible for its nuclear export under desensitization conditions.

Interestingly, we observed that the interaction between AKT and Mdm2 increased under desensitization conditions; however, this response was not detected when the K8/14R-AKT mutant was used (Fig. 7B), which cannot undergo ubiquitination (Fig. 2C). As shown by immunocytochemistry (Fig. 7C), AKT and Mdm2 were detected mainly in the nucleus or on the plasma membrane in the resting state when co-expressed with D_3_. In response to repeated exposure to D_3_R agonists, some AKT translocated to the cell membrane and co-localized with Mdm2, whereas K8/14R-AKT did not. Furthermore, Mdm2 was found to be phosphorylated at serine residue(s) under desensitization conditions, and this modification was blocked by pretreatment with triciribine (Fig. 7D) or knockdown of TRAF6 (Fig. 7E). Additionally, when co-expressing D_3_R, Mdm2 was observed to localize to the cell membrane under resting and non-desensitizing conditions; however, upon repeated agonist exposure, Mdm2 was recruited to the nucleus (Fig. 7F), which is consistent with previous reports [10, 11]. Knocking down of TRAF6 abolished D_3_R desensitization condition-induced nuclear entry of Mdm2 (Fig. 7F).

**Fig. 8:**
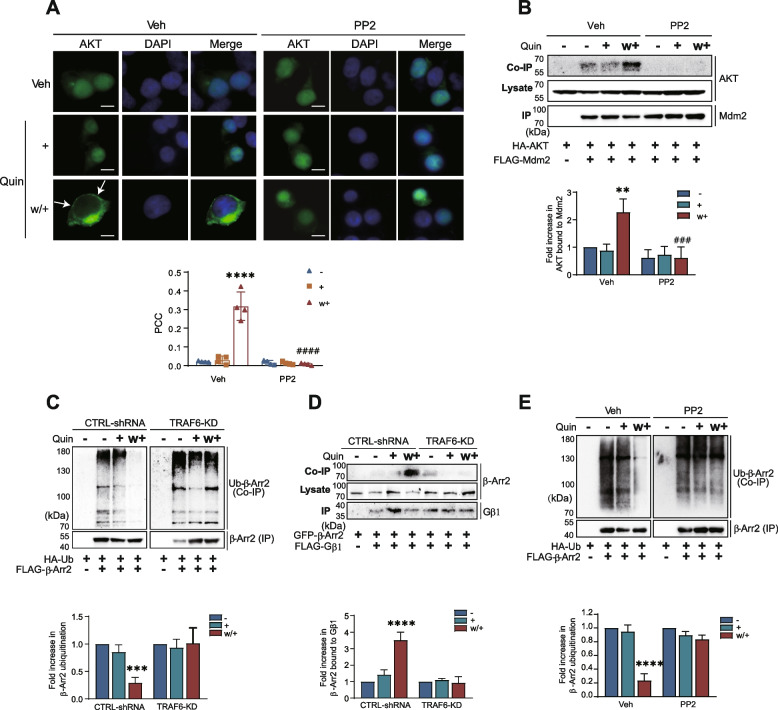
Roles of AKT ubiquitination in β-arrestin2 deubiquitination under desensitization conditions. **A** Cells expressing D_3_R were transfected with GFP-AKT. Cells were pretreated with 10 μM PP2 for 30 min, then treated with 100 nM Quin for 5 min, washed, and re-challenged. The scale bar represents 10 μm. Membrane localization of AKT was determined by Pearson correlation coefficient. Data show one representative example out of three independent experiments. ****p < 0.0001 compared to CTRL-shRNA/Veh group, ^####^*p* < 0.0001 compared to CTRL-shRNA/w + group (*n* = 4). **B** HEK293 cells expressing D_3_R were transfected with FLAG-Mdm2 and HA-AKT. Cells were pretreated with 10 μM PP2 for 30 min, then treated with 100 nM Quin for 5 min, washed, and re-challenged with Quin for 5 min. **p < 0.01 compared to Veh/veh group, ^###^*p* < 0.001 compared to Veh/w + group (*n* = 3). **C** CTRL-shRNA and TRAF6-KD cells were transfected with D_3_R, HA-Ub, and FLAG-β-Arr2. Then the cells were treated with 100 nM Quin for 5 min, washed, and re-challenged. ***p < 0.001 compared to “CTRL-shRNA/−” group (*n* = 3). **D** CTRL-shRNA and TRAF6-KD cells were transfected with D_3_R, GFP-β-Arr2, and FLAG-Gβ1. Then the cells were treated with 100 nM Quin for 5 min, washed, and re-challenged with Quin for 5 min. ****p < 0.0001 compared to “CTRL-shRNA/−” group (*n* = 3). **E** HEK293 cells expressing D_3_R were transfected with HA-Ub and FLAG-β-Arr2. The cells were pretreated with either vehicle or 10 μM PP2 for 30 min, followed by treatment with 100 nM Quin for 5 min, washed, and re-challenged with Quin for 5 min. ****p < 0.0001 compared to “Veh/−” group (*n* = 3)

Consistent with the inhibition of AKT ubiquitination by pretreatment with PP2 (Fig. 6B), this pretreatment was found to block AKT nuclear export (Fig. 8A) and the interaction of AKT with Mdm2 (Fig. 8B). Thus, it can be postulated that AKT ubiquitination is necessary for its interaction with Mdm2, which subsequently mediates its phosphorylation at S166/S186 and facilitates its nuclear entry.

### Ubiquitinated AKT is responsible for β-Arr2 deubiquitination, which sequesters Gβγ under desensitization conditions

The recruitment of Mdm2 to the nucleus has been proposed to be responsible for the deubiquitination of β-arrestin, which induces receptor desensitization by sequestering Gβγ [11]. Pretreatment with the AKT inhibitor triciribine abolishes the deubiquitination of β-Arr2 under desensitization conditions [17]. We tested whether AKT ubiquitination was required for this process. Under desensitization conditions, deubiquitination of β-Arr2 (Fig. 8C) and a concomitant increase in the interaction between Gβγ and β-Arr2 (Fig. 8D) occurred in a TRAF6-dependent manner. Pretreatment with PP2, which inhibited AKT ubiquitination (Fig. 6B), also blocked the deubiquitination of β-Arr2 (Fig. 8E). These findings indicate that repeated agonist exposure facilitates AKT ubiquitination, leading to the deubiquitination of β-Arr2. Subsequently, deubiquitinated β-Arr2 forms a complex with Gβγ, which contributes to receptor desensitization.

### Desensitization of β_2_ adrenergic receptors can be explained by the TRAF6-mediated ubiquitination of AKT

To confirm whether the desensitization mechanism of D_3_R and D_2_R variant observed in this study applies to other receptors, we investigated the β_2_ adrenergic receptor (β_2_AR), which has been widely used as a study model to characterize the molecular mechanisms of GPCRs desensitization, which underlying that β-arrestin-mediated physical uncoupling between receptors and G proteins serves as the fundamental mechanism of GPCR desensitization.

**Fig. 9:**
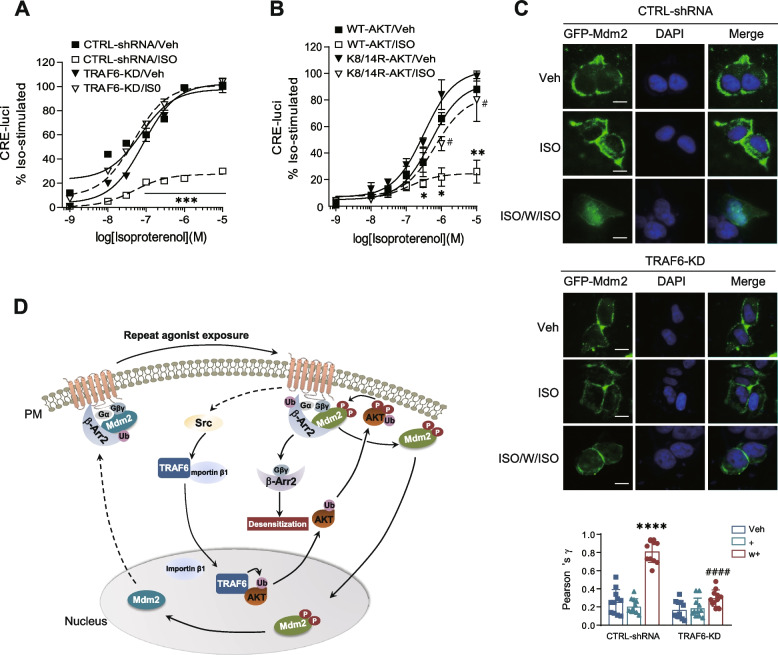
AKT ubiquitination is required for β_2_AR desensitization. **A** CTRL-shRNA and TRAF6-KD cells were transfected with β_2_AR (1.5 ~ 1.7 pmol/mg protein). Then the cells were pretreated with vehicle or 1 μM ISO for 5 min, washed three times, and treated with increasing concentrations of ISO. The cellular levels of cAMP were evaluated by using the CRE-luciferase reporter gene assay. CTRL-shRNA/ISO group was significantly different from other groups at treatment concentrations of ISO between 10^−7^ and 10^−5^ M. ***p < 0.001 compared to “CTRL-shRNA/ Veh” group (*n* = 3). **B** HEK293 cells were cotransfected with β_2_AR, WT-AKT or K8/14R-AKT. Then the cells were pre-treated with vehicle or 1 μM ISO 5 min, washed three times, and treated with increasing concentrations of ISO. The cellular levels of cAMP were evaluated by using the CRE-luciferase reporter gene assay. The WT-AKT/ISO group was significantly different from the WT-AKT/Veh group at isoproterenol concentrations between 3 × 10^−7^ and 10^−5^ M (*p < 0.05, **p < 0.01 (*n* = 3)). The K8/14R-AKT/ISO group was significantly different from the K8/14R-AKT/Veh at isoproterenol concentrations between 10^−6^ and 10^−5^ M (^#^*p* < 0.05 (*n* = 3)). **C** CTRL-shRNA and TRAF6-KD cells were cotransfected with β_2_AR and GFP-Mdm2. The cells were treated with 1 μM ISO for 2 min, washed, and re-challenged. The colocalization between Mdm2 and DAPI is shown as Pearson’s coefficient. ****p < 0.0001 compared to corresponding “CTRL-shRNA/Veh” group, ^####^*p* < 0.0001 compared to “CTRL-shRNA/w+” group (*n* = 11). The scale bar represents 10 μm. **D** Diagram showing the molecular mechanisms of AKT ubiquitination in homologous desensitization of GPCRs: Under desensitization conditions (repeated agonist exposure), TRAF6 interacts with importin β1 and enters the nucleus in a Src-dependent manner. Within the nucleus, TRAF6-mediated the ubiquitination of AKT, thereby promoting its the translocation and activation. Phosphorylated AKT subsequently phosphorylates Mdm2, which is in complex with the receptor, Gβγ and β-Arr2. Thereafter, phosphorylated Mdm2 is recruited to the nucleus, leading to the de-ubiquitination of β-Arr2. Deubiquitinated β-Arr2 then forms a complex with Gβγ, leading to the desensitization of GPCRs

As shown in Fig. 9A and B, knocking down TRAF6 or co-expressing K8/14R-AKT abolished the desensitization of β_2_AR. These results suggest that the desensitization principle of D_3_R and D_2_R variant, which involves TRAF6-mediated ubiquitination of AKT, obtained from this study is also applicable to the desensitization of β_2_AR. Furthermore, the nuclear entry of Mdm2 was abolished upon TRAF6 knockdown via repeated exposure to a β_2_AR agonist (Fig. 9C). Overall, our findings suggest that the mechanism of D_3_R and D_2_R variant desensitization is likely applicable to the desensitization of other GPCRs.

## Discussion

The binding of β-arrestins to the transmembrane core of phosphorylated GPCRs induces steric hindrance, precluding further G protein coupling, and has been widely accepted as the classical model for GPCRs homologous desensitization [32]. Unfortunately, several studies have reported that the conventional regulatory model is not applicable for explaining homologous desensitization of some GPCRs, such as D_2_-like receptors and parathyroid hormone receptors [10, 11, 33]. Our study reveals a novel regulatory mechanism underlying the desensitization of D_3_R and D_2_R variant and, to some extent, β_2_AR receptor. The major findings are summarized in Fig. 9D. In the resting state, receptors prone to desensitization are constitutively associated with Mdm2 within the plasma membrane in a Gβγ- and β-arrestin-dependent manner, where β-arrestin is in ubiquitination state [10, 21]. Repeated agonist exposure initiates a signaling cascade that employs Src tyrosine kinase to mediate the nuclear entry of TRAF6 in an importin β1-dependent manner (Figs. 5 & 6), promoting AKT ubiquitination in the nucleus (Figs. 3 & 4). Subsequently, ubiquitinated AKT translocates to the plasma membrane (Figs. 3C, 7A & 8A) and is phosphorylated at residues T308 and S473, leading to its full activation (Figs. 1 & 2). Activated AKT interacts with Mdm2 and phosphorylates it at the S166 and S186 residues, thereby promoting deubiquitination of β-arrestin2 by accelerating nuclear entry of Mdm2 (Figs. 7 & 8). Deubiquitinated β-arrestin2 forms a complex with Gβγ (Fig. 8D), sequestering Gβγ from the receptors and inhibiting receptor signaling transduction [11, 17].

Our previous data showed that 1 min to 5 min agonist treatment can initiate D_3_R desensitization, with a 5 min treatment inducing maximal desensitization (data not shown). Repeated exposure of cells expressing D_3_R to quinpirole for 1 min or 5 min resulted in the dissociation of D_3_R from Gαo (data not shown). These findings indicate that D_3_R desensitization occurs rapidly, becoming apparent after just 1 min of repeated agonist exposure, and approaches near-maximum levels within 5 min. It is important to note that D_3_R does not undergo agonist-induced internalization. Therefore, to elucidate the mechanism of D_3_R desensitization more clearly, we opted for a 5 min agonist treatment to ensure maximal desensitization. In addition, the time courses of D_3_R desensitization, TRAF6 nuclear entry, and AKT nuclear export coincide. 2 min treatment with quinpirole facilitated TRAF6 nuclear entry (Fig. 5A) and AKT nuclear export (Fig. 7A), supporting the temporal correlation between these processes.

A recent study revealed that AKT regulates the desensitization of D_2_-like receptors by phosphorylating the deubiquitinase USP33 [17]. In this work, we demonstrated that activated AKT promotes GPCRs desensitization through its interaction with Mdm2. Thus, it could be speculated that AKT exerts two distinct effects on the homologous desensitization of GPCRs: one mechanism involves AKT regulating receptor desensitization by promoting the nuclear entry of Mdm2, while an alternative mechanism involves the promotion of the interaction of USP33 with β-arrestin. Notably, we showed that ubiquitination of AKT is a key cellular event that triggers its functional role in GPCRs desensitization through the aforementioned mechanisms.

Ubiquitin is attached to target proteins through an enzymatic cascade, starting with an activating enzyme (E1) that is charged with ubiquitin in an ATP-dependent process. The ubiquitin molecule is then transferred to an E2-conjugating enzyme and ultimately attached to a target protein by an E3 ligase, which determines target specificity [34]. Ubiquitination of AKT is a general event that can be triggered by various growth factors and cytokines, including insulin-like growth factor 1 (IGF-1), epidermal growth factor (EGF), and interleukin-1. Different E3 ligases have been shown to be involved in mediating AKT ubiquitination in response to different extracellular stimuli. For example, Skp2-SCF specifically engages in AKT ubiquitination triggered by the activation of ErbB family receptors. NEDD4–1 is responsible for the ubiquitination of AKT in response to IGF-1 stimulation. However, TRAF6 has also been reported to act as an E3 ligase enzyme responsible for IGF-1-induced AKT ubiquitination [22, 35, 36]. In this study, we demonstrated that TRAF6 is the key E3 ligase responsible for AKT ubiquitination under GPCRs desensitization conditions.

Although ubiquitination has traditionally been viewed as a critical marker for protein degradation [37], several studies have revealed that ubiquitination also exerts non-proteolytic effects. For example, protein ubiquitination plays a functional role in promoting the endocytosis of receptor tyrosine kinases by recruiting components of the internalization machinery [38]. Ubiquitination can also regulate the activity of transcription factors by recruiting coactivators/corepressors or by mediating their nuclear export [39, 40]. In this study, we demonstrated for the first time that ubiquitination of AKT triggers its nuclear export under desensitization conditions. In addition, ubiquitination can function as a nuclear import trigger. For example, nuclear entry of the transcription factor NF-κB is indirectly regulated by ubiquitination [41]. Ubiquitination of the E2-conjugating enzymes UbcM2, UbcH6 and UBE2E2 promotes their interaction with importin 11, resulting in their nuclear entry [42].

TRAF6, a member of the TNFR family, regulates numerous protein molecules through both ubiquitination and non-ubiquitination mechanisms. It has been shown that TRAF6 regulates the kinase activity of TAK1 by acting as an E3 ubiquitin ligase [43]. TRAF6 binds to the p75 cytoplasmic interactor NRIF, and subsequently promotes its nuclear localization [44], and it also mediates the activation of NF-κB by interacting with aPKC [45]. Furthermore, TRAF6 has been reported to interact with Src and increase its kinase activity [30]. However, in our study, Src kinase was shown to regulate the nuclear entry of TRAF6 by promoting its interaction with importin β1, which seems to be the initial event in mediating GPCRs desensitization. In fact, the functional roles of Src tyrosine kinase in the development of certain GPCRs have been reported. For example, Src mediates the desensitization of β_2_AR by phosphorylating both GRK2 and receptor [46]. Additionally, Src promotes δ-opioid receptor desensitization by interfering with receptor endocytosis [47]. On the basis of study, it could be assumed that TRAF6, Src and importin β1 might translocate to the nucleus as a tri-complex under desensitization conditions. Indeed, the tyrosine kinase Src has been shown to translocate into the nucleus, although there is a lack of nuclear localization signal (NLS) [48]. However, examining how the kinase activity of Src increases and how Src subcellularly localizes under receptor desensitization conditions will be of particular interest.

The interplay between ubiquitination and phosphorylation has emerged as prominent posttranslational crosstalk in eukaryotic cell signaling [49]. Phosphorylation often serves as a marker that triggers subsequent ubiquitination [50, 51]. This phenomenon has been observed in various cases, such as with the androgen receptor [52], c-Myc [53] or the yeast transcriptional factor Rpn4 [54]. However, TRAF6-mediated K63-linked ubiquitination of IRF7 reportedly preludes its phosphorylation, which is required for LMP1-stimulated transcriptional activity [55, 56]. Here, we identified two key lysine residues (K8 and K14) that are required for AKT phosphorylation and activation. Mutation of these sites results in a decrease in ubiquitination and abrogates the desensitization of D_3_R, D_2_R variant and β_2_AR. Our findings with this ubiquitination-deficient mutant imply that ubiquitination of AKT precedes its phosphorylation (Fig. 2). It is well known that AKT undergoes K63-linked ubiquitination [22]. Thus, K48-linked ubiquitination is typically preceded by phosphorylation. However, K63-linked ubiquitination occurs independently of phosphorylation.

Beaulieu et al.’s work elegantly demonstrates the role of β-arrestin in DA-dependent AKT inactivation. Their study emphasized the complex interplay between AKT, β-arrestin, and PP2A, promoting certain positive aspects of DA receptor signaling [57]. However, it has also been shown that the absence of β-Arr2 may enhance DA receptor signaling and the behavioral response to DA [58]. These findings suggest that β-arrestin may exert contradictory roles in modulating GPCRs signaling. Our study, in line with previous research, proposes that the modification of β-arrestin ubiquitination status is a pivotal cellular event underlying receptor desensitization [59]. Accordingly, it is posited that ubiquitination generally enables β-arrestin to effectively associate with GPCRs, along with other proteins and mechanisms involved in receptor signaling. Conversely, deubiquitination of arrestin can alter the cellular environments optimized for signaling, thereby diminishing signaling efficiency.

The present study proposes a novel regulatory mechanism for GPCRs desensitization that differs from that of the conventional regulatory model, which highlights the critical role of β-arrestin-induced steric hindrance. This novel signaling cascade involves Src, TRAF6, ubiquitinated AKT, and recruited of Mdm2 into the nucleus, where it mediates the deubiquitination of β-arrestin, ultimately resulting in receptor desensitization. The mechanistic principles established in this study based on studies of D_3_R, D_2_R variant and β_2_AR could be employed to predict the desensitization of other GPCRs and offer novel methods for manipulating desensitization. Given the functional and pathological implications of GPCRs desensitization, the results of this study are critical for both fundamental research and clinical investigations.

### Supplementary Information

**Supplementary Material 1.**

## Data Availability

No datasets were generated or analysed during the current study.

## Abbreviations

AKT: Protein kinase B
β_2_AR: β_2_ Adrenergic receptor
DA: Dopamine
D_3_R: Dopamine D_3_ receptor
GRK2: GPCR kinase 2
GPCRs: G protein-coupled receptors
IP: Immunoprecipitates
ISO: Isoproterenol
Quin: Quinpirole
SDS-PAGE: Sodium dodecyl sulfate polyacrylamide gel electrophoresis
TRAF6: TNF receptor-associated factor 6

## References

[1] Williams JT, Ingram SL, Henderson G, Chavkin C, von Zastrow M, Schulz S (2013). Regulation of mu-opioid receptors: desensitization, phosphorylation, internalization, and tolerance. Pharmacol Rev.

[2] Ferguson SS (2001). Evolving concepts in G protein-coupled receptor endocytosis: the role in receptor desensitization and signaling. Pharmacol Rev.

[3] Thomsen ARB, Plouffe B, Cahill TJ, Shukla AK, Tarrasch JT, Dosey AM (2016). GPCR-G protein-beta-Arrestin super-complex mediates sustained G protein signaling. Cell..

[4] Pao CS, Benovic JL (2002). Phosphorylation-independent desensitization of G protein-coupled receptors?. Sci STKE.

[5] Carman CV, Parent JL, Day PW, Pronin AN, Sternweis PM, Wedegaertner PB (1999). Selective regulation of Galpha(q/11) by an RGS domain in the G protein-coupled receptor kinase, GRK2. J Biol Chem.

[6] Ribeiro FM, Ferreira LT, Paquet M, Cregan T, Ding Q, Gros R, Ferguson SS (2009). Phosphorylation-independent regulation of metabotropic glutamate receptor 5 desensitization and internalization by G protein-coupled receptor kinase 2 in neurons. J Biol Chem.

[7] Kim K-M, Valenzano KJ, Robinson SR, Yao WD, Barak LS, Caron MG (2001). Differential regulation of the dopamine D2and D3 receptors by G protein-coupled receptor kinases and β-arrestins. J Biol Chem.

[8] Cho D-I, Beom S, Van Tol HH, Caron MG, Kim K-M (2006). Characterization of the desensitization properties of five dopamine receptor subtypes and alternatively spliced variants of dopamine D2 and D4 receptors. Biochem Biophys Res Commun.

[9] Min C, Zheng M, Zhang X, Caron M, Kim K (2013). Novel roles for β-arrestins in the regulation of pharmacological sequestration to predict agonist-induced desensitization of dopamine D3 receptors. Br J Pharmacol.

[10] Zheng M, Zhang X, Sun N, Min X, Acharya S, Kim K-M (2020). A novel molecular mechanism responsible for phosphorylation-independent desensitization of G protein-coupled receptors exemplified by the dopamine D3 receptor. Biochem Biophys Res Commun.

[11] Zheng M, Zhang X, Min X, Sun N, Kim K-M (2020). Cytoplasmic recruitment of Mdm2 as a common characteristic of G protein-coupled receptors that undergo desensitization. Biochem Biophys Res Commun.

[12] Zhang X, Sun N, Zheng M, Kim K-M (2016). Clathrin-mediated endocytosis is responsible for the lysosomal degradation of dopamine D3 receptor. Biochem Biophys Res Commun.

[13] Liu H, Ma H, Zeng X, Wu C, Acharya S, Sudan SK, Zhang X. Ubiquitination of GRK2 is required for the beta-Arrestin-biased signaling pathway of dopamine D2 receptors to activate ERK kinases. Int J Mol Sci. 2023;24(12). 10.3390/ijms241210031 PMC10298151 37373182

[14] Cho D, Zheng M, Min C, Ma L, Kurose H, Park JH, Kim KM (2010). Agonist-induced endocytosis and receptor phosphorylation mediate resensitization of dopamine D(2) receptors. Mol Endocrinol.

[15] Min X, Zhang X, Sun N, Acharya S, Kim KM (2019). Mdm2-mediated ubiquitination of PKCbetaII in the nucleus mediates clathrin-mediated endocytic activity. Biochem Pharmacol.

[16] Pan W, Jia Y, Wang J, Tao D, Gan X, Tsiokas L (2005). Beta-catenin regulates myogenesis by relieving I-mfa-mediated suppression of myogenic regulatory factors in P19 cells. Proc Natl Acad Sci USA.

[17] Min X, Sun N, Wang S, Zhang X, Kim KM (2023). Sequestration of Gbetagamma by deubiquitinated arrestins into the nucleus as a novel desensitization mechanism of G protein-coupled receptors. Cell Commun Signal.

[18] Sarbassov DD, Guertin DA, Ali SM, Sabatini DM (2005). Phosphorylation and regulation of Akt/PKB by the rictor-mTOR complex. Science..

[19] Westrich L, Kuzhikandathil EV (2007). The tolerance property of human D3 dopamine receptor is determined by specific amino acid residues in the second cytoplasmic loop. Biochim Biophys Acta.

[20] Westrich L, Gil-Mast S, Kortagere S, Kuzhikandathil EV (2010). Development of tolerance in D3 dopamine receptor signaling is accompanied by distinct changes in receptor conformation. Biochem Pharmacol.

[21] Min C, Zhang X, Zheng M, Sun N, Acharya S, Zhang X, Kim KM (2017). Molecular signature that determines the acute tolerance of G protein-coupled receptors. Biomol Ther (Seoul).

[22] Yang WL, Wang J, Chan CH, Lee SW, Campos AD, Lamothe B (2009). The E3 ligase TRAF6 regulates Akt ubiquitination and activation. Science..

[23] Wang R, Brattain MG (2006). AKT can be activated in the nucleus. Cell Signal.

[24] Pham LV, Zhou HJ, Lin-Lee YC, Tamayo AT, Yoshimura LC, Fu L (2008). Nuclear tumor necrosis factor receptor-associated factor 6 in lymphoid cells negatively regulates c-Myb-mediated transactivation through small ubiquitin-related modifier-1 modification. J Biol Chem.

[25] Kudo N, Wolff B, Sekimoto T, Schreiner EP, Yoneda Y, Yanagida M (1998). Leptomycin B inhibition of signal-mediated nuclear export by direct binding to CRM1. Exp Cell Res.

[26] Meier R, Alessi DR, Cron P, Andjelkovic M, Hemmings BA (1997). Mitogenic activation, phosphorylation, and nuclear translocation of protein kinase Bbeta. J Biol Chem.

[27] Shiraishi I, Melendez J, Ahn Y, Skavdahl M, Murphy E, Welch S (2004). Nuclear targeting of Akt enhances kinase activity and survival of cardiomyocytes. Circ Res.

[28] Sekimoto T, Yoneda Y (2012). Intrinsic and extrinsic negative regulators of nuclear protein transport processes. Genes Cells.

[29] Zhang X, Min X, Wang S, Sun N, Kim KM (2020). Mdm2-mediated ubiquitination of beta-arrestin2 in the nucleus occurs in a Gbetagamma- and clathrin-dependent manner. Biochem Pharmacol.

[30] Wang J, Wu X, Jiang M, Tai G (2020). Mechanism by which TRAF6 participates in the immune regulation of autoimmune diseases and Cancer. Biomed Res Int.

[31] Mayo LD, Donner DB (2001). A phosphatidylinositol 3-kinase/Akt pathway promotes translocation of Mdm2 from the cytoplasm to the nucleus. Proc Natl Acad Sci USA.

[32] Cahill TJ, Thomsen AR, Tarrasch JT, Plouffe B, Nguyen AH, Yang F (2017). Distinct conformations of GPCR-beta-arrestin complexes mediate desensitization, signaling, and endocytosis. Proc Natl Acad Sci USA.

[33] Wehbi VL, Stevenson HP, Feinstein TN, Calero G, Romero G, Vilardaga JP (2013). Noncanonical GPCR signaling arising from a PTH receptor-arrestin-Gbetagamma complex. Proc Natl Acad Sci USA.

[34] Pickart CM (2001). Mechanisms underlying ubiquitination. Annu Rev Biochem.

[35] Chan CH, Li CF, Yang WL, Gao Y, Lee SW, Feng Z (2012). The Skp2-SCF E3 ligase regulates Akt ubiquitination, glycolysis, herceptin sensitivity, and tumorigenesis. Cell..

[36] Fan CD, Lum MA, Xu C, Black JD, Wang X (2013). Ubiquitin-dependent regulation of phospho-AKT dynamics by the ubiquitin E3 ligase, NEDD4-1, in the insulin-like growth factor-1 response. J Biol Chem.

[37] Ciechanover A, Ben-Saadon R (2004). N-terminal ubiquitination: more protein substrates join in. Trends Cell Biol.

[38] Marmor MD, Yarden Y (2004). Role of protein ubiquitylation in regulating endocytosis of receptor tyrosine kinases. Oncogene..

[39] Muratani M, Tansey WP (2003). How the ubiquitin-proteasome system controls transcription. Nat Rev Mol Cell Biol.

[40] Shcherbik N, Haines DS (2004). Ub on the move. J Cell Biochem.

[41] Ghosh S, Karin M (2002). Missing pieces in the NF-kappaB puzzle. Cell..

[42] Plafker SM, Plafker KS, Weissman AM, Macara IG (2004). Ubiquitin charging of human class III ubiquitin-conjugating enzymes triggers their nuclear import. J Cell Biol.

[43] Wang C, Deng L, Hong M, Akkaraju GR, Inoue J, Chen ZJ (2001). TAK1 is a ubiquitin-dependent kinase of MKK and IKK. Nature..

[44] Geetha T, Kenchappa RS, Wooten MW, Carter BD (2005). TRAF6-mediated ubiquitination regulates nuclear translocation of NRIF, the p75 receptor interactor. EMBO J.

[45] Sanz L, Diaz-Meco MT, Nakano H, Moscat J (2000). The atypical PKC-interacting protein p62 channels NF-kappaB activation by the IL-1-TRAF6 pathway. EMBO J.

[46] Fan G, Shumay E, Malbon CC, Wang H (2001). C-Src tyrosine kinase binds the beta 2-adrenergic receptor via phospho-Tyr-350, phosphorylates G-protein-linked receptor kinase 2, and mediates agonist-induced receptor desensitization. J Biol Chem.

[47] Hong MH, Xu C, Wang YJ, Ji JL, Tao YM, Xu XJ (2009). Role of Src in ligand-specific regulation of delta-opioid receptor desensitization and internalization. J Neurochem.

[48] Bagnato G, Leopizzi M, Urciuoli E, Peruzzi B. Nuclear functions of the tyrosine kinase Src. Int J Mol Sci. 2020;21(8). 10.3390/ijms21082675 PMC7215861 32290470

[49] Hunter T (2007). The age of crosstalk: phosphorylation, ubiquitination, and beyond. Mol Cell.

[50] Fuchs SY, Dolan L, Davis RJ, Ronai Z (1996). Phosphorylation-dependent targeting of c-Jun ubiquitination by Jun N-kinase. Oncogene..

[51] Magnani M, Crinelli R, Bianchi M, Antonelli A (2000). The ubiquitin-dependent proteolytic system and other potential targets for the modulation of nuclear factor-kB (NF-kB). Curr Drug Targets.

[52] Lin HK, Wang L, Hu YC, Altuwaijri S, Chang C (2002). Phosphorylation-dependent ubiquitylation and degradation of androgen receptor by Akt require Mdm2 E3 ligase. EMBO J.

[53] Yada M, Hatakeyama S, Kamura T, Nishiyama M, Tsunematsu R, Imaki H (2004). Phosphorylation-dependent degradation of c-Myc is mediated by the F-box protein Fbw7. EMBO J.

[54] Ju D, Xu H, Wang X, Xie Y (2007). Ubiquitin-mediated degradation of Rpn4 is controlled by a phosphorylation-dependent ubiquitylation signal. Biochim Biophys Acta.

[55] Huye LE, Ning S, Kelliher M, Pagano JS (2007). Interferon regulatory factor 7 is activated by a viral oncoprotein through RIP-dependent ubiquitination. Mol Cell Biol.

[56] Ning S, Campos AD, Darnay BG, Bentz GL, Pagano JS (2008). TRAF6 and the three C-terminal lysine sites on IRF7 are required for its ubiquitination-mediated activation by the tumor necrosis factor receptor family member latent membrane protein 1. Mol Cell Biol.

[57] Beaulieu J-M, Sotnikova TD, Marion S, Lefkowitz RJ, Gainetdinov RR, Caron MG (2005). An Akt/β-arrestin 2/PP2A signaling complex mediates dopaminergic neurotransmission and behavior. Cell..

[58] Gainetdinov RR, Caron MG (2003). Monoamine transporters: from genes to behavior. Annu Rev Pharmacol Toxicol.

[59] Min X, Sun N, Wang S, Zhang X, Kim K-M (2023). Sequestration of Gβγ by deubiquitinated arrestins into the nucleus as a novel desensitization mechanism of G protein–coupled receptors. Cell Commun Signaling.

