# Supplementary material for: TRAF6-mediated ubiquitination of AKT in the nucleus is a critical event underlying the desensitization of G protein-coupled receptors
Source: Cell Commun Signal. 2024 Apr 2;22:213. doi: 10.1186/s12964-024-01592-z (PMC10986131; doi:10.1186/s12964-024-01592-z)
Supplement: Supplementary file 1 — Supplementary Material 1. [file 12964_2024_1592_MOESM1_ESM.pdf]

Fig.1B

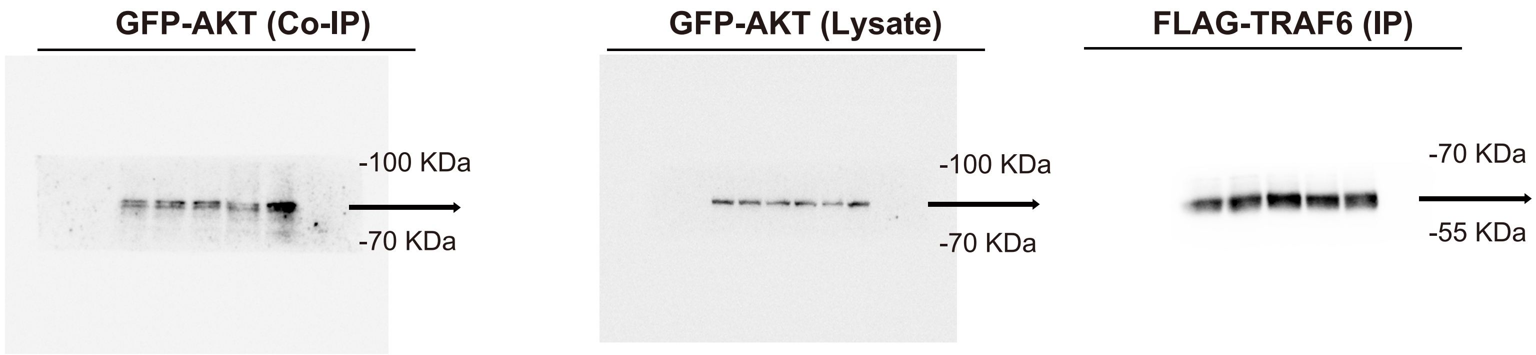
GFP-AKT (Co-IP)
-100 KDa
-70 KDa
GFP-AKT (Lysate)
-100 KDa
-70 KDa
FLAG-TRAF6 (IP)
-70 KDa
-55 KDa

Fig.1C

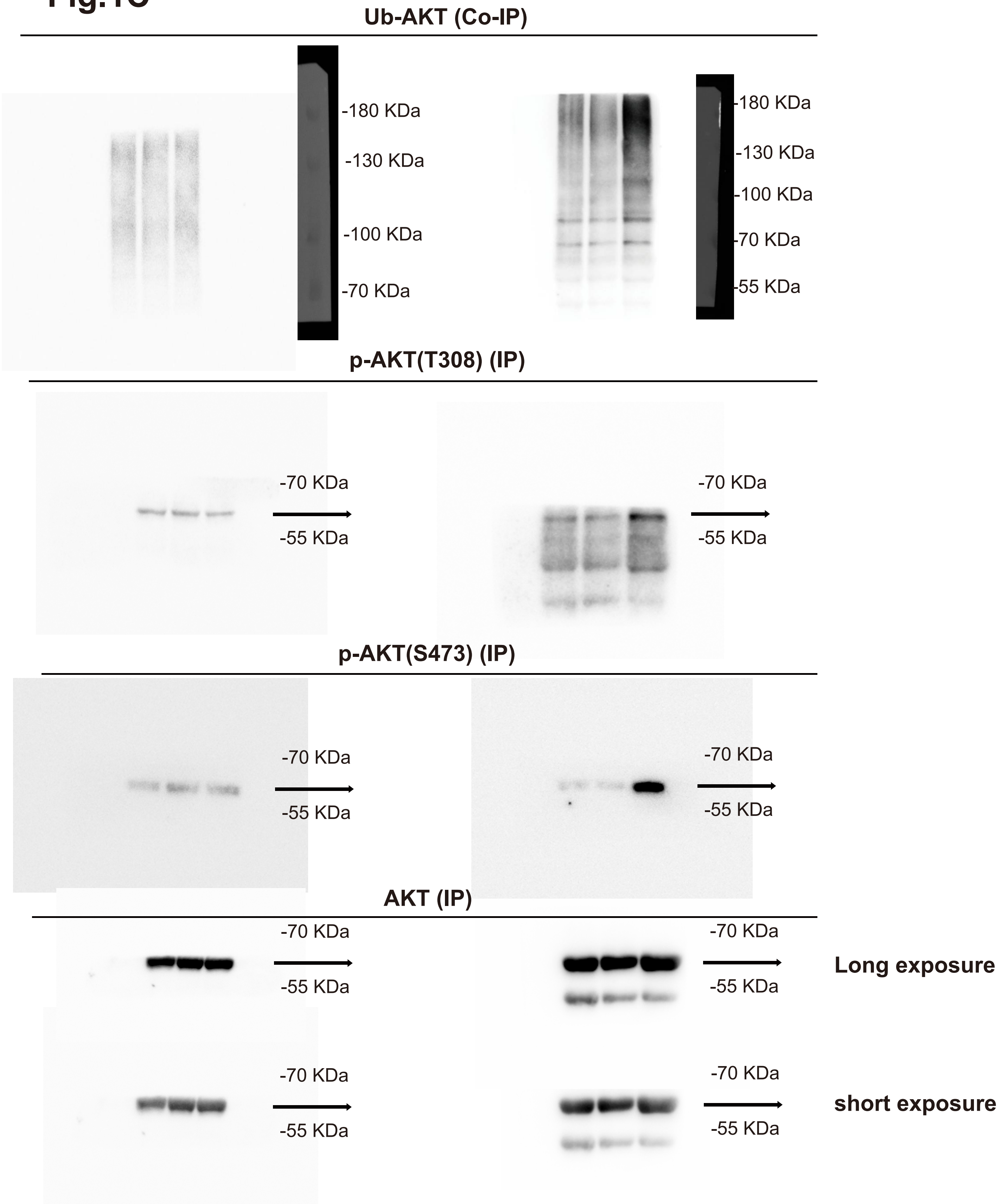
Ub-AKT (Co-IP)
-180 KDa
-130 KDa
-100 KDa
-70 KDa
-180 KDa
-130 KDa
-100 KDa
-70 KDa
-55 KDa
p-AKT(T308) (IP)
-70 KDa
-55 KDa
-70 KDa
-55 KDa
p-AKT(S473) (IP)
-70 KDa
-55 KDa
-70 KDa
-55 KDa
AKT (IP)
-70 KDa
-55 KDa
-70 KDa
-55 KDa
Long exposure
-70 KDa
-55 KDa
-70 KDa
-55 KDa
short exposure

Fig.1D

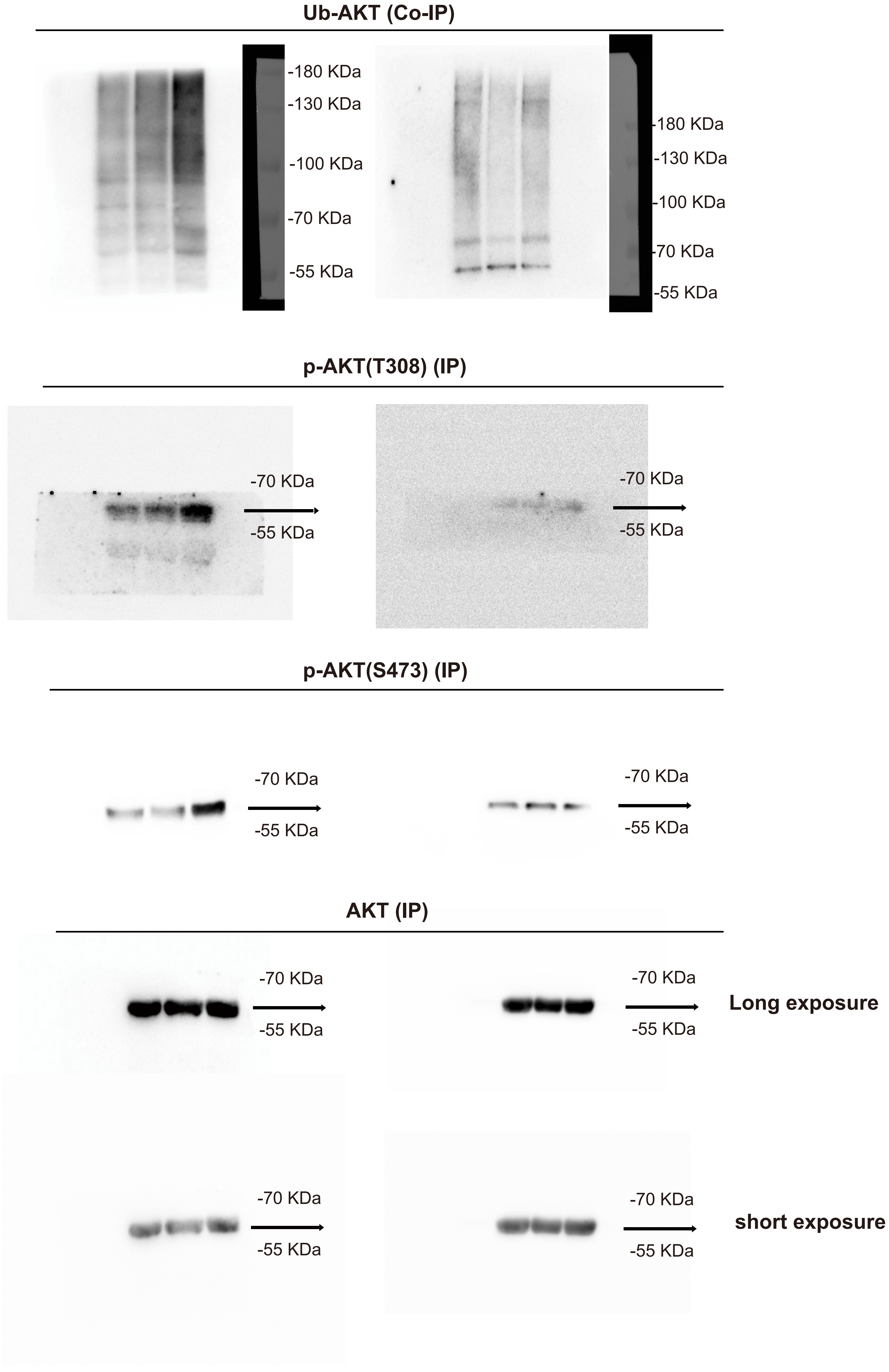
Ub-AKT (Co-IP)
-180 KDa
-130 KDa
-100 KDa
-70 KDa
-55 KDa
-180 KDa
-130 KDa
-100 KDa
-70 KDa
-55 KDa
p-AKT(T308) (IP)
-70 KDa
-55 KDa
-70 KDa
-55 KDa
p-AKT(S473) (IP)
-70 KDa
-55 KDa
-70 KDa
-55 KDa
AKT (IP)
-70 KDa
-55 KDa
-70 KDa
-55 KDa
Long exposure
-70 KDa
-55 KDa
-70 KDa
-55 KDa
short exposure

Fig.1E

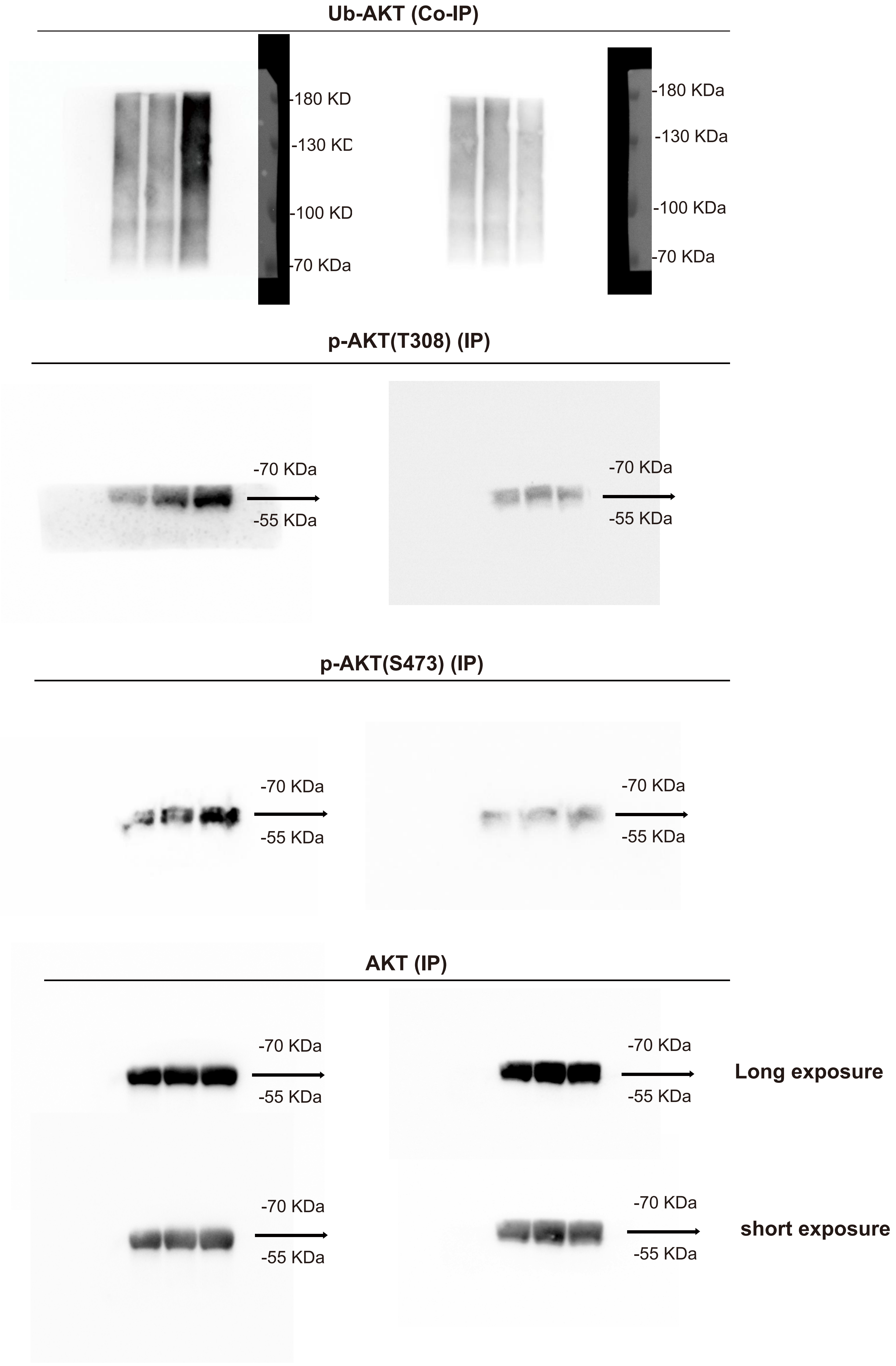
Ub-AKT (Co-IP)
-180 KD
-130 KD
-100 KD
-70 KDa
-180 KDa
-130 KDa
-100 KDa
-70 KDa
p-AKT(T308) (IP)
-70 KDa
-55 KDa
-70 KDa
-55 KDa
p-AKT(S473) (IP)
-70 KDa
-55 KDa
-70 KDa
-55 KDa
AKT (IP)
-70 KDa
-55 KDa
-70 KDa
-55 KDa
Long exposure
-70 KDa
-55 KDa
-70 KDa
-55 KDa
short exposure

Fig.1F

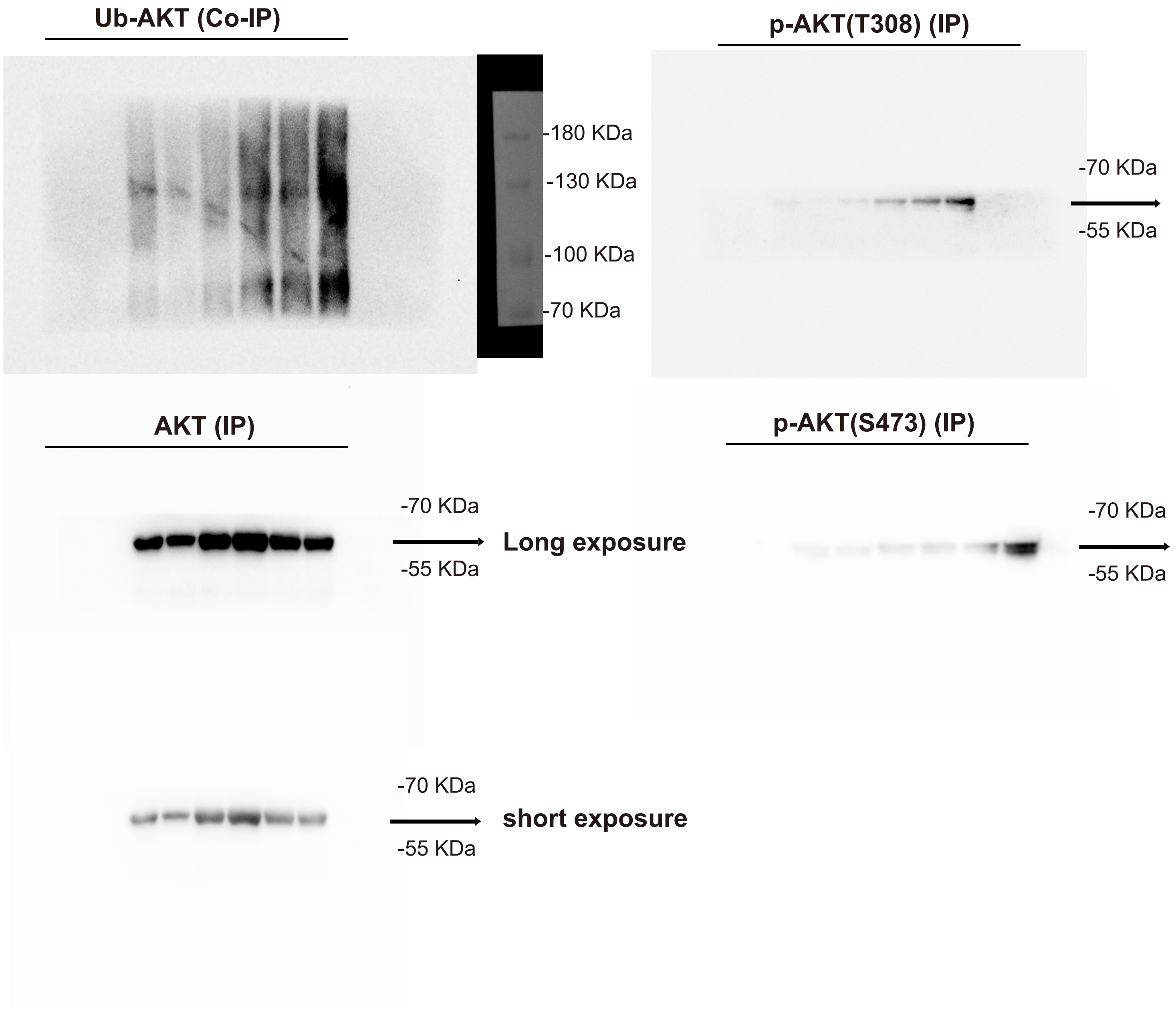
Ub-AKT (Co-IP)
-180 KDa
-130 KDa
-100 KDa
-70 KDa
p-AKT(T308) (IP)
-70 KDa
-55 KDa
AKT (IP)
-70 KDa
Long exposure
-55 KDa
p-AKT(S473) (IP)
-70 KDa
-55 KDa
-70 KDa
short exposure
-55 KDa

Fig.2A

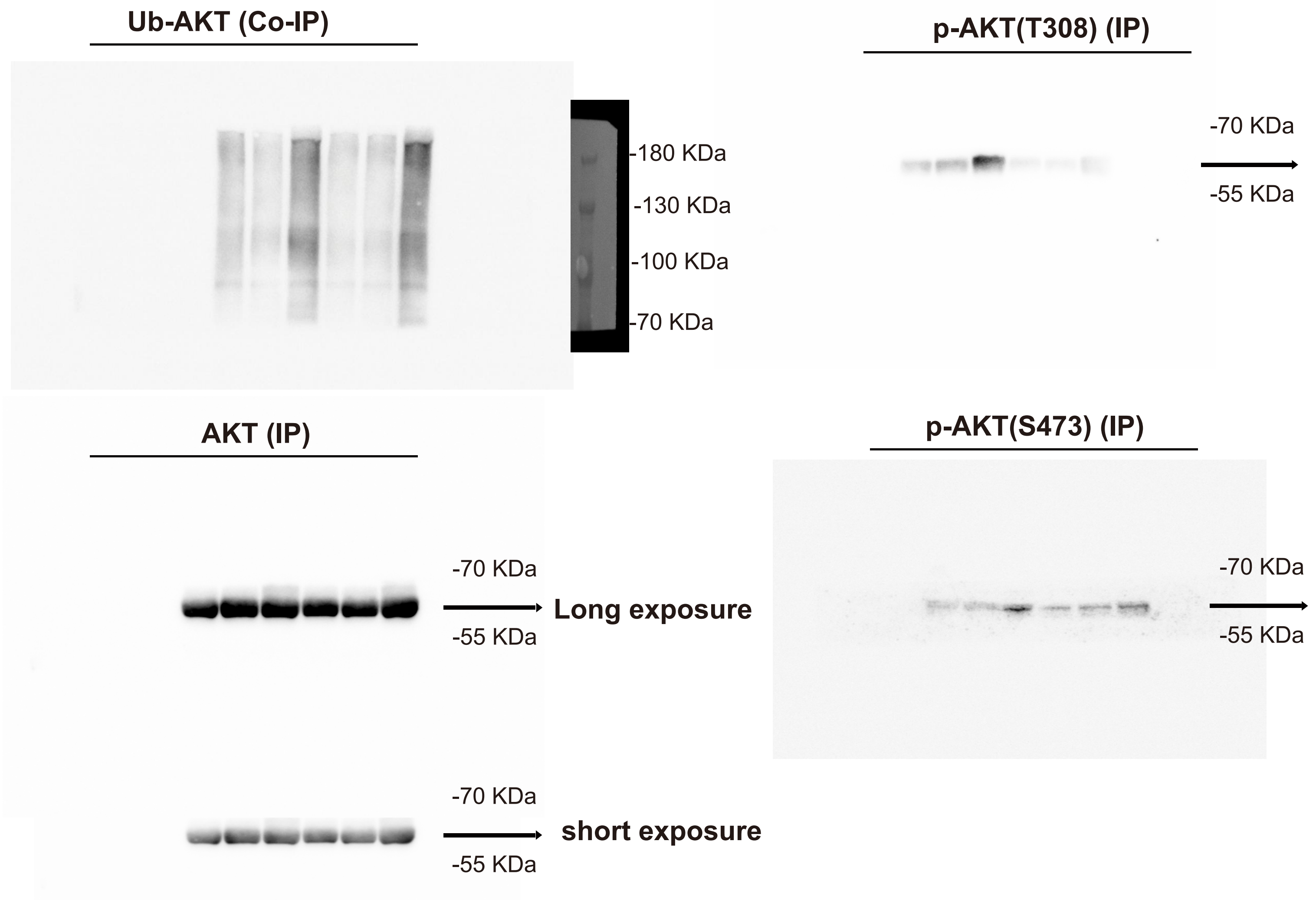
Ub-AKT (Co-IP)
-180 KDa
-130 KDa
-100 KDa
-70 KDa
p-AKT(T308) (IP)
-70 KDa
-55 KDa
AKT (IP)
-70 KDa
-55 KDa
Long exposure
-70 KDa
-55 KDa
short exposure
p-AKT(S473) (IP)
-70 KDa
-55 KDa

Fig.2B

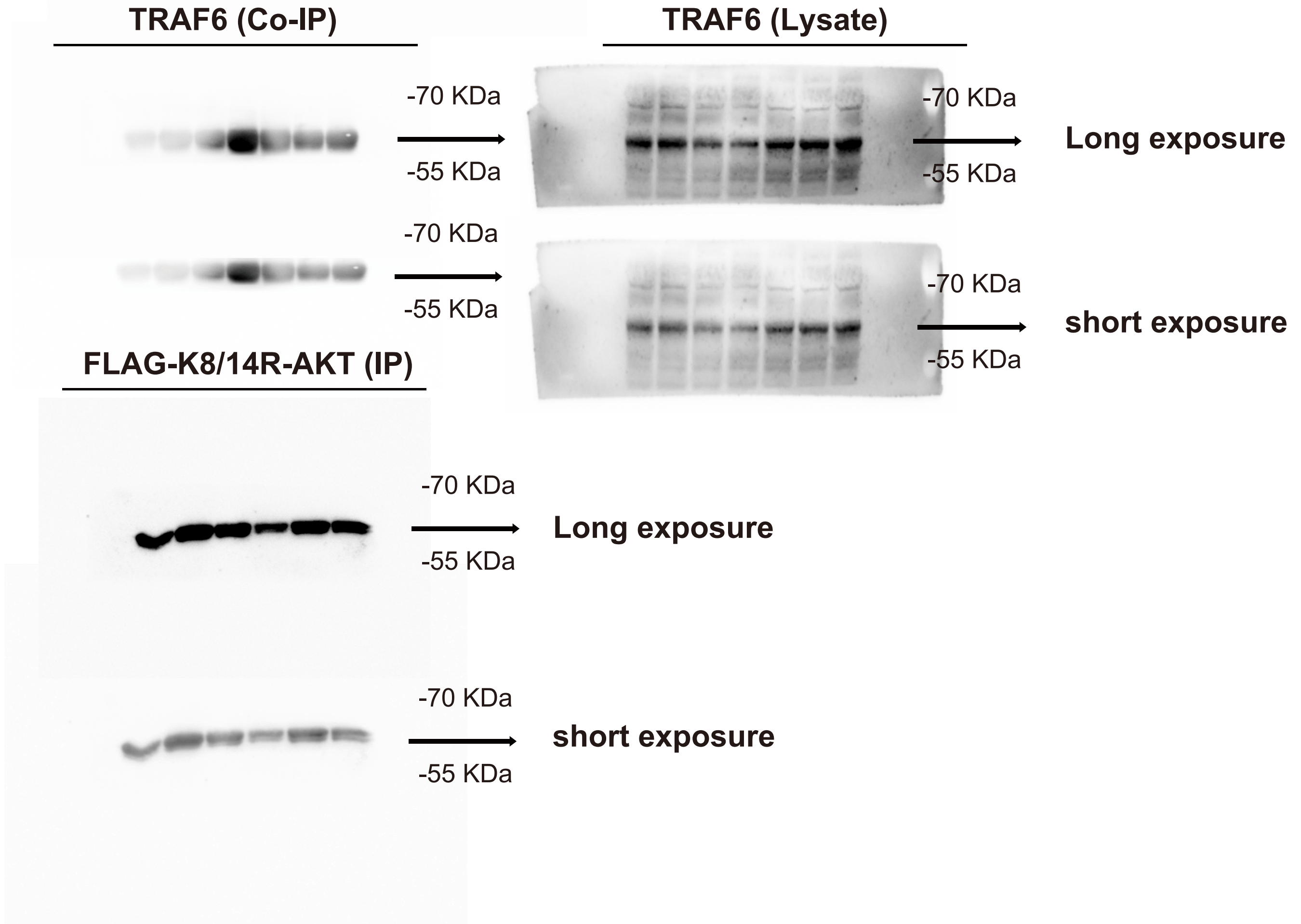
TRAF6 (Co-IP)
TRAF6 (Lysate)
-70 KDa
-55 KDa
-70 KDa
-55 KDa
Long exposure
-70 KDa
-55 KDa
-70 KDa
-55 KDa
short exposure
FLAG-K8/14R-AKT (IP)
-70 KDa
-55 KDa
Long exposure
-70 KDa
-55 KDa
short exposure

Fig.2C

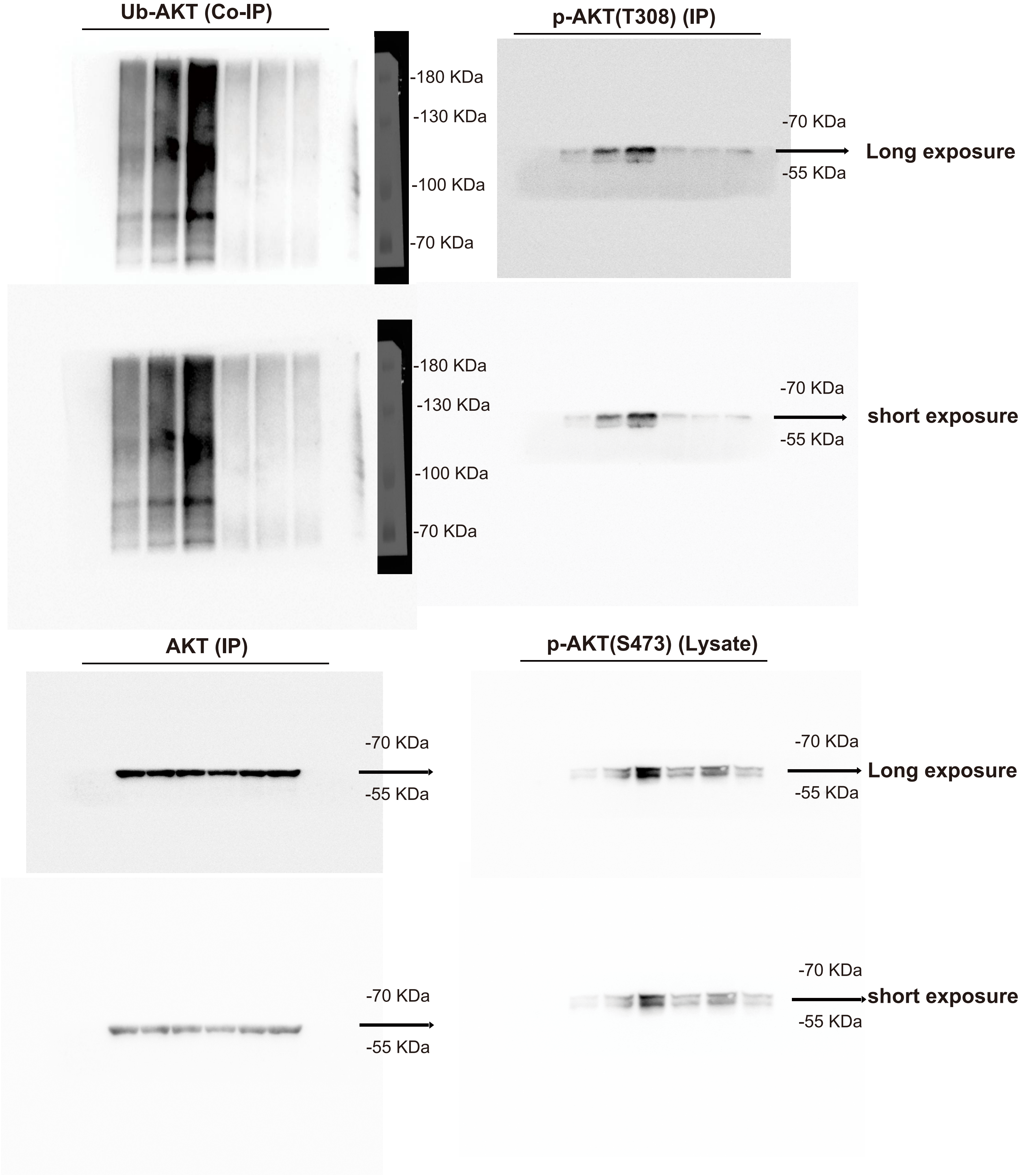

Ub-AKT (Co-IP)
-180 KDa
-130 KDa
-100 KDa
-70 KDa
p-AKT(T308) (IP)
-70 KDa
-55 KDa
Long exposure
-180 KDa
-130 KDa
-100 KDa
-70 KDa
-70 KDa
-55 KDa
short exposure
AKT (IP)
-70 KDa
-55 KDa
p-AKT(S473) (Lysate)
-70 KDa
-55 KDa
Long exposure
-70 KDa
-55 KDa
-70 KDa
-55 KDa
short exposure

Fig.3E

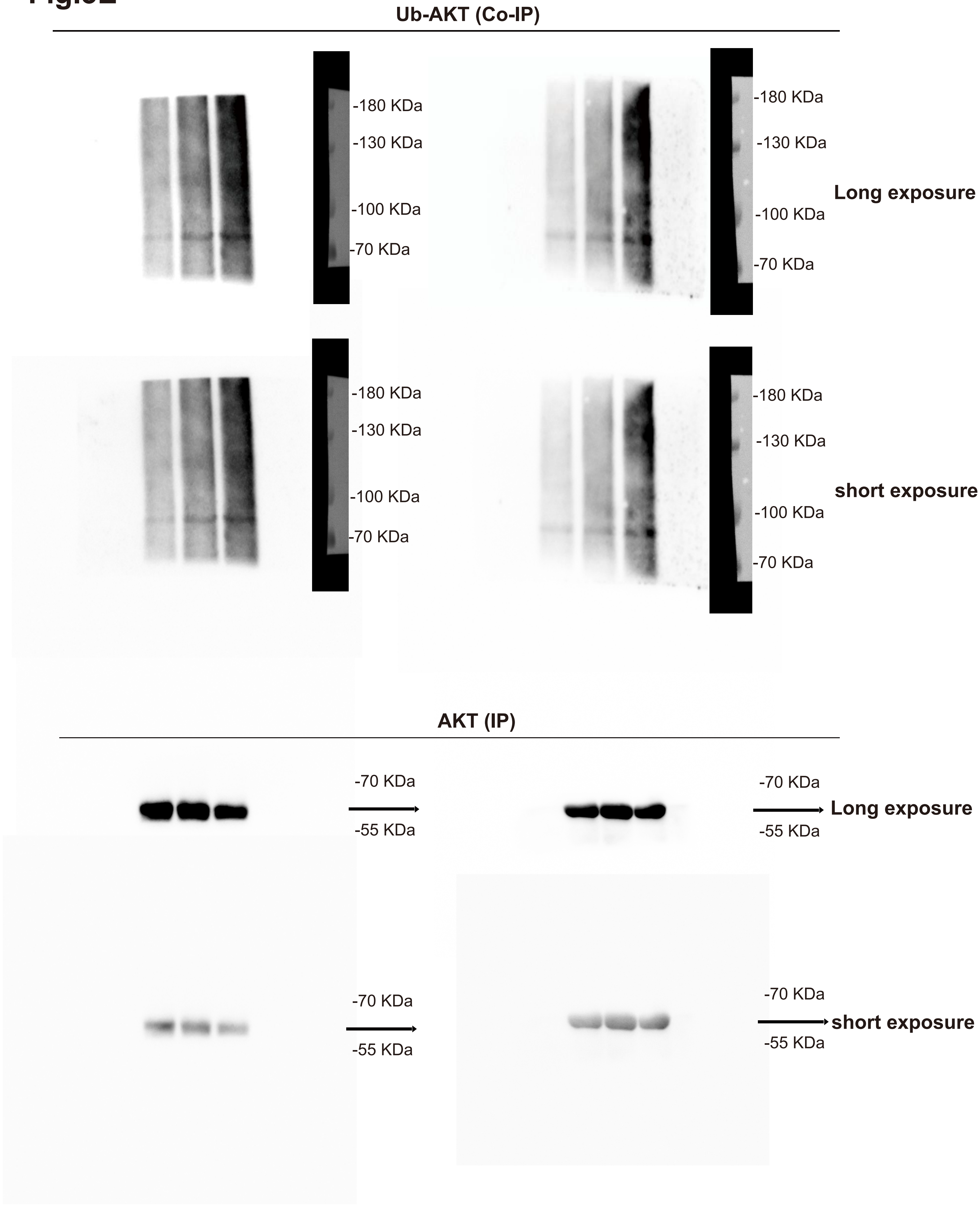

Ub-AKT (Co-IP)
-180 KDa
-130 KDa
-100 KDa
-70 KDa
-180 KDa
-130 KDa
-100 KDa
-70 KDa
Long exposure
-180 KDa
-130 KDa
-100 KDa
-70 KDa
-180 KDa
-130 KDa
-100 KDa
-70 KDa
short exposure
AKT (IP)
-70 KDa
-55 KDa
-70 KDa
-55 KDa
Long exposure
-70 KDa
-55 KDa
-70 KDa
-55 KDa
short exposure

Fig.4A

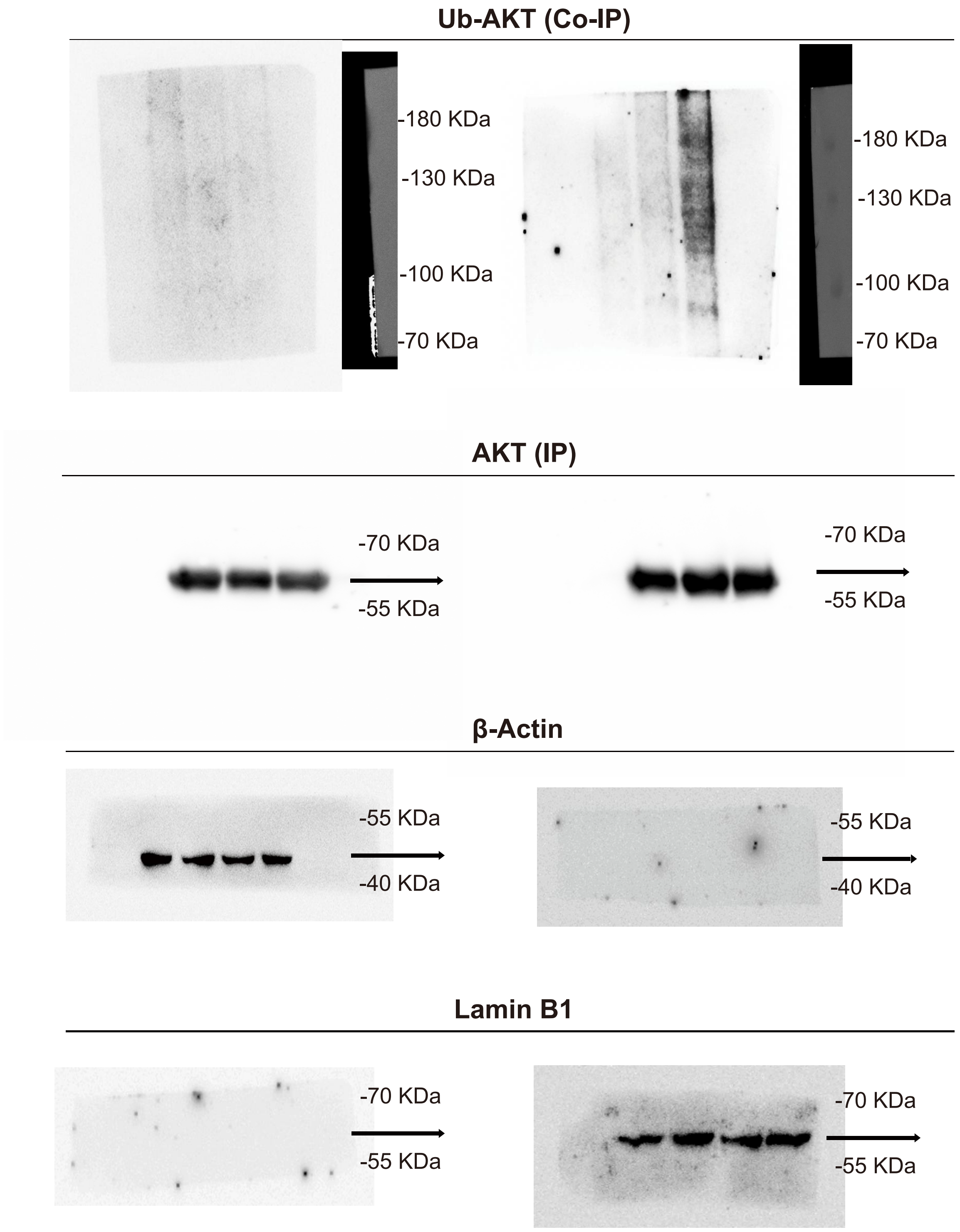
Ub-AKT (Co-IP)
-180 KDa
-130 KDa
-100 KDa
-70 KDa
-180 KDa
-130 KDa
-100 KDa
-70 KDa
AKT (IP)
-70 KDa
-55 KDa
-70 KDa
-55 KDa
β-Actin
-55 KDa
-40 KDa
-55 KDa
-40 KDa
Lamin B1
-70 KDa
-55 KDa
-70 KDa
-55 KDa

Fig.4B

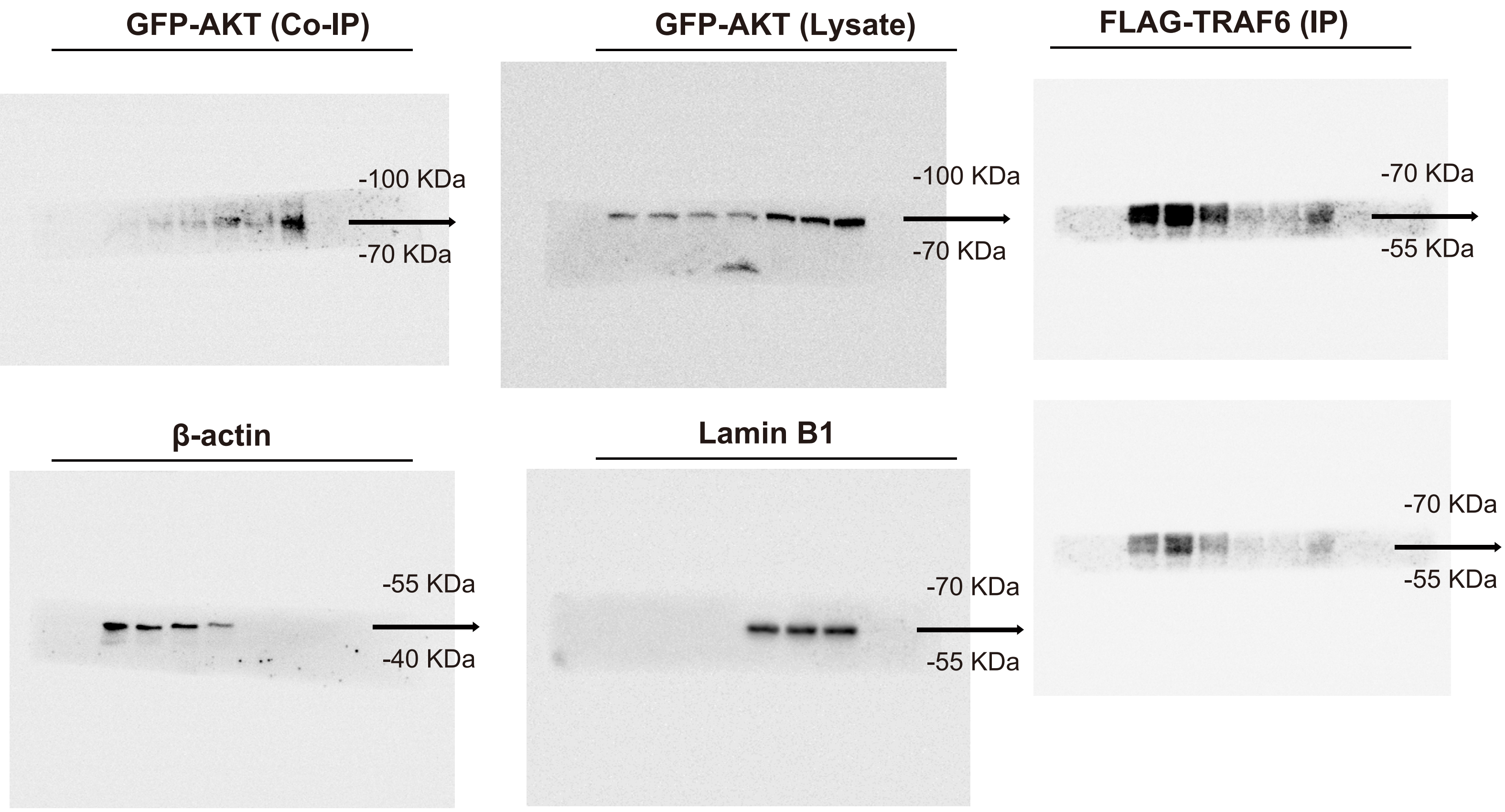
GFP-AKT (Co-IP)
-100 KDa
-70 KDa
GFP-AKT (Lysate)
-100 KDa
-70 KDa
FLAG-TRAF6 (IP)
-70 KDa
-55 KDa
β-actin
-55 KDa
-40 KDa
Lamin B1
-70 KDa
-55 KDa
-70 KDa
-55 KDa

Fig.4E

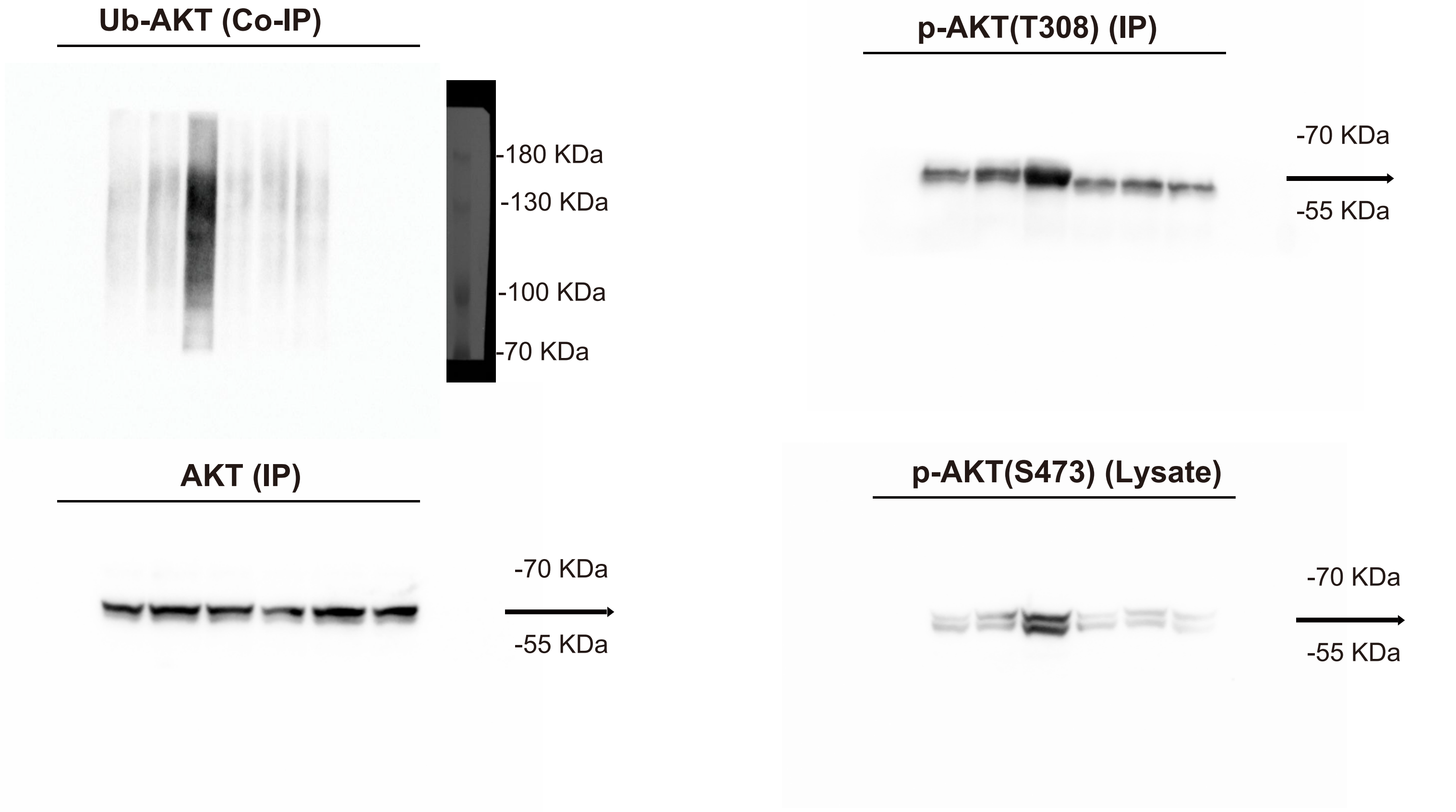
Ub-AKT (Co-IP)
-180 KDa
-130 KDa
-100 KDa
-70 KDa
p-AKT(T308) (IP)
-70 KDa
-55 KDa
AKT (IP)
-70 KDa
-55 KDa
p-AKT(S473) (Lysate)
-70 KDa
-55 KDa

Fig.5D

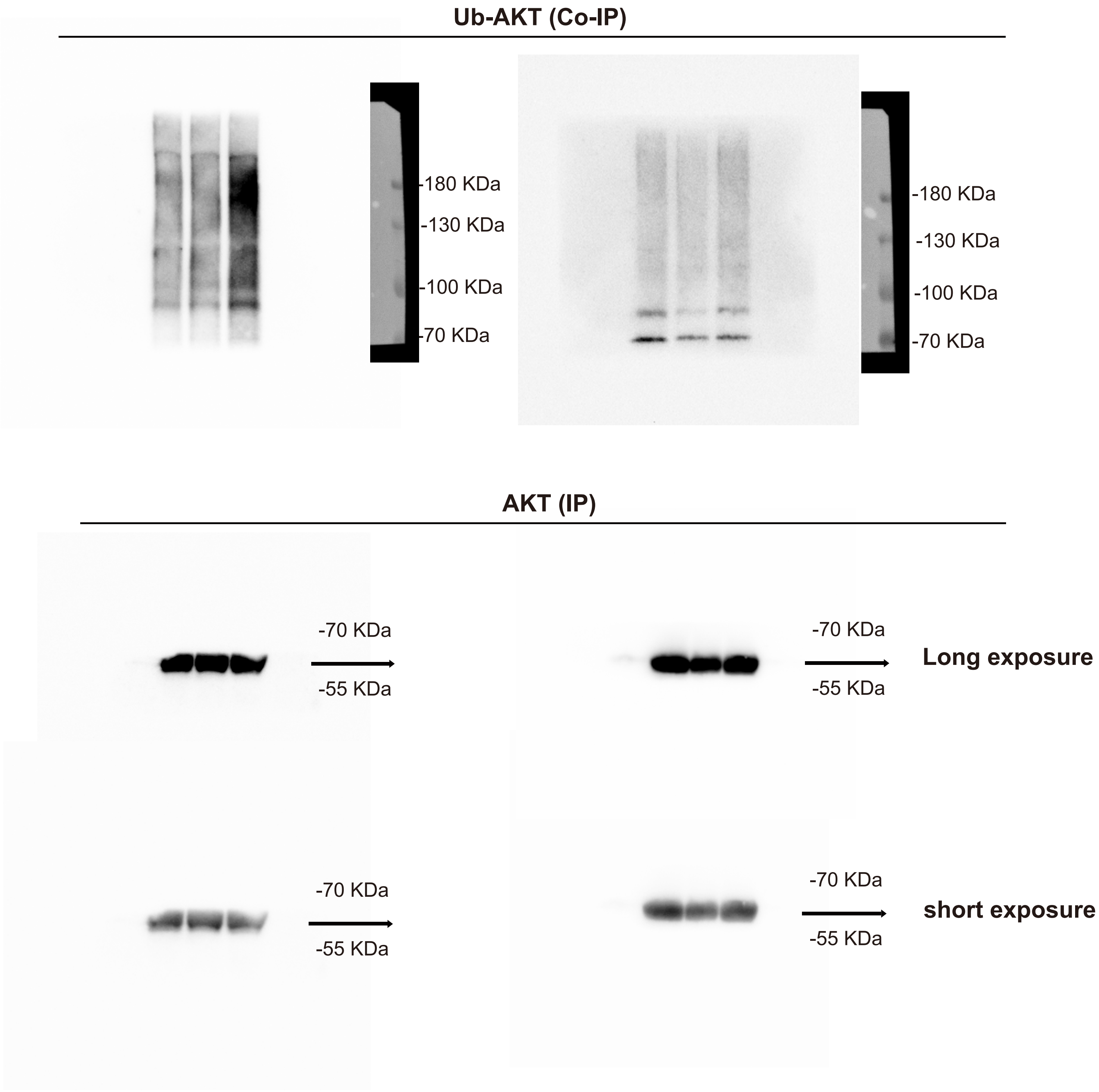
Ub-AKT (Co-IP)
-180 KDa
-130 KDa
-100 KDa
-70 KDa
-180 KDa
-130 KDa
-100 KDa
-70 KDa
AKT (IP)
-70 KDa
-55 KDa
-70 KDa
-55 KDa
Long exposure
-70 KDa
-55 KDa
-70 KDa
-55 KDa
short exposure

Fig.6B

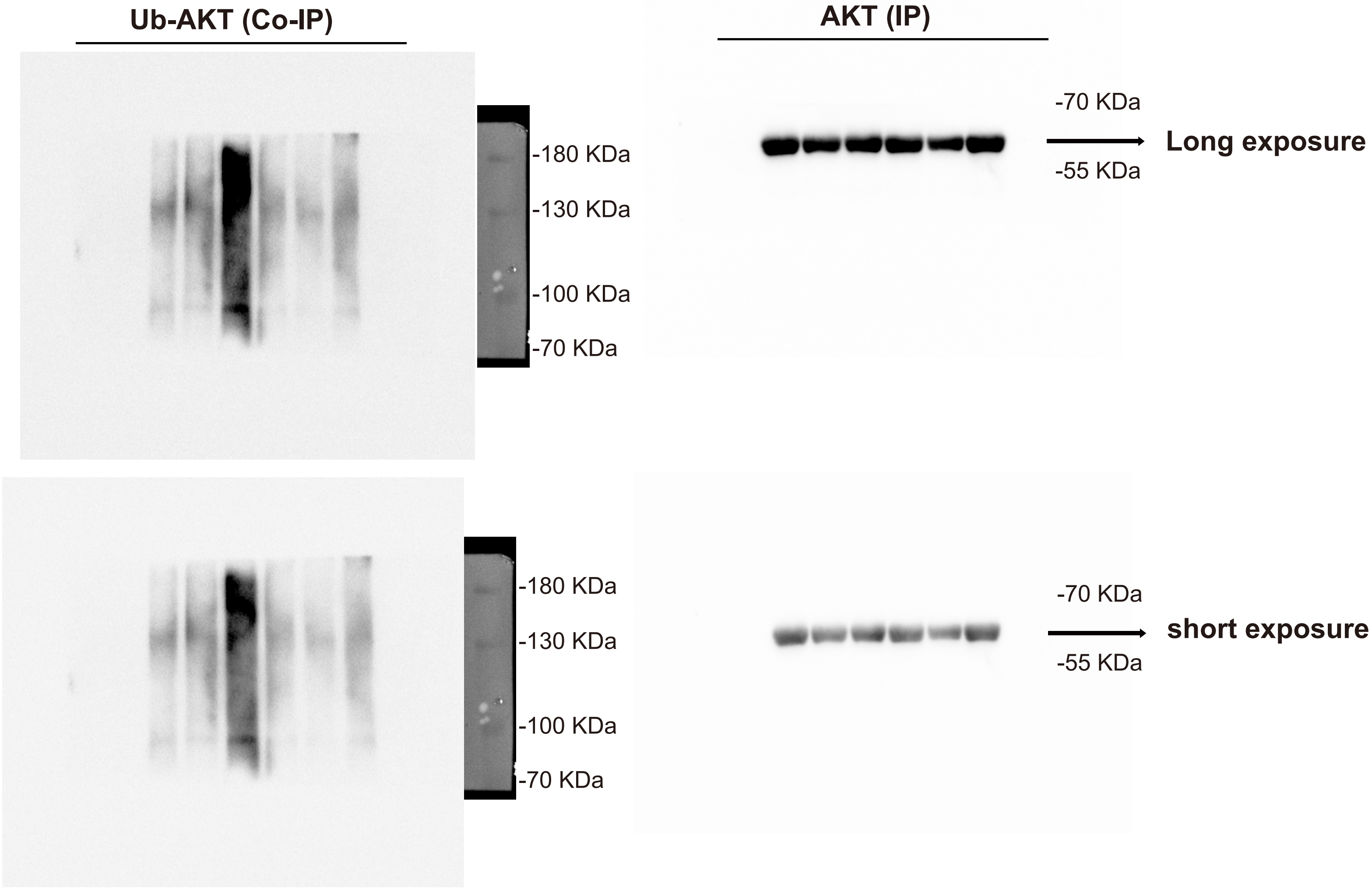
Ub-AKT (Co-IP)
AKT (IP)
-180 KDa
-130 KDa
-100 KDa
-70 KDa
-70 KDa
-55 KDa
Long exposure
-180 KDa
-130 KDa
-100 KDa
-70 KDa
-70 KDa
-55 KDa
short exposure

Fig.6C

GFP-AKT (Co-IP)

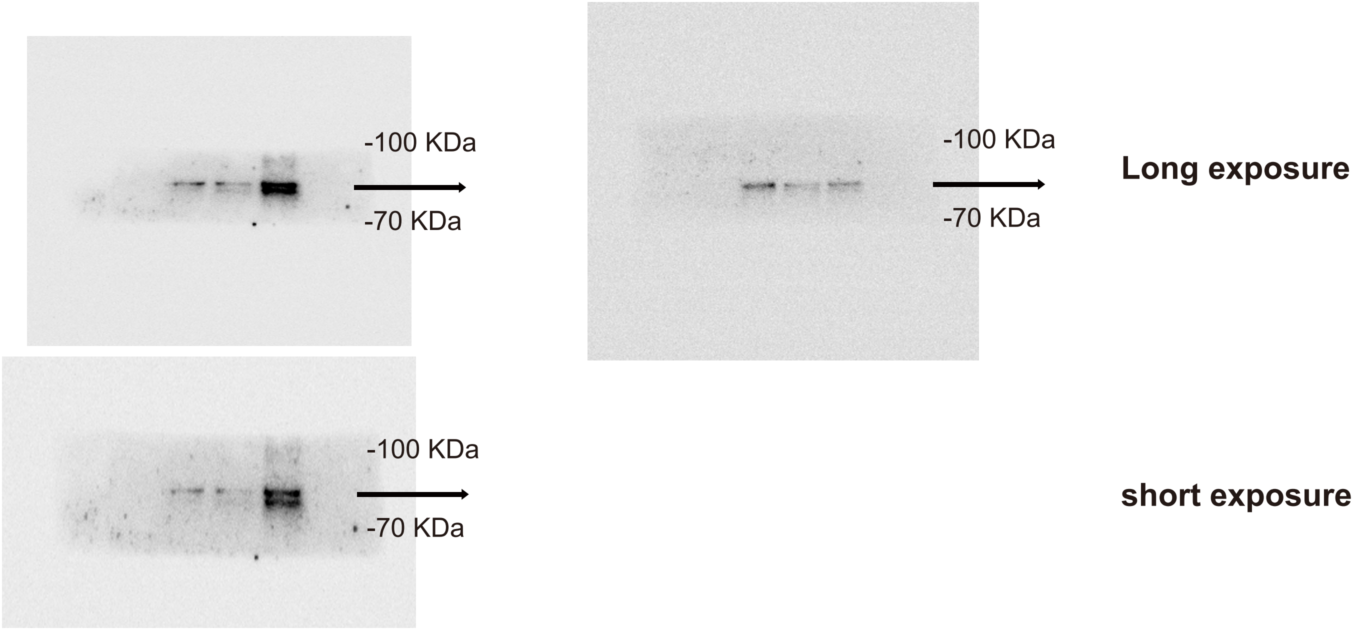

GFP-AKT (Lysate)

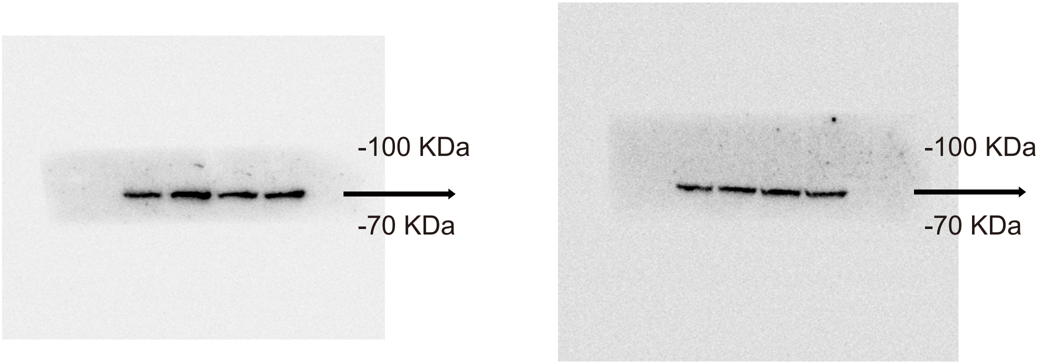

FLAG-TRAF6 (IP)

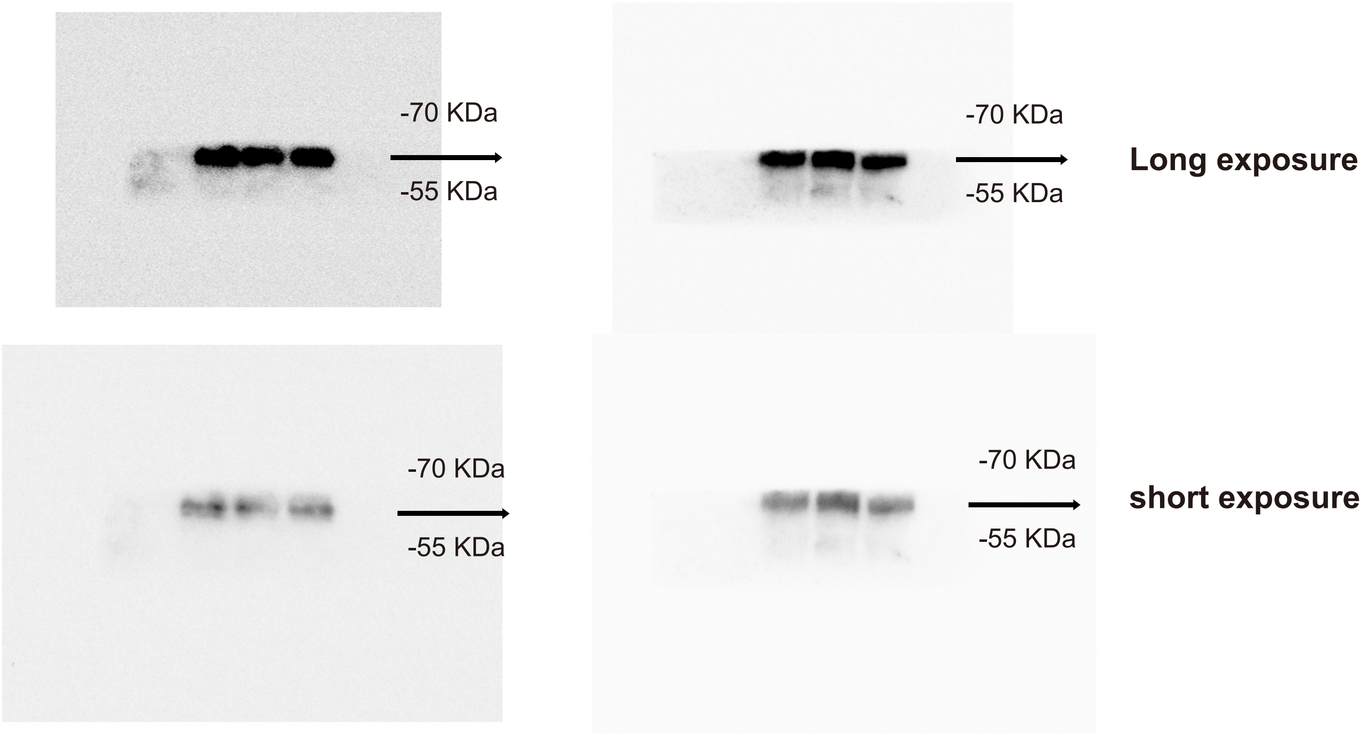

Fig.6D

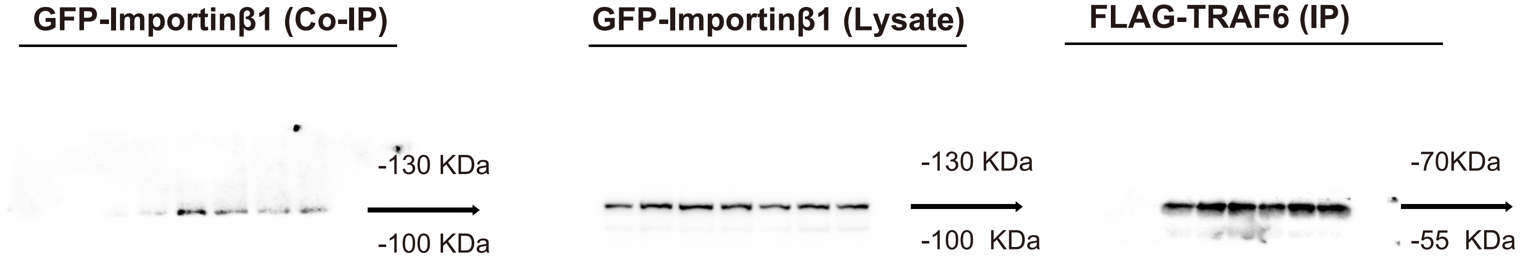
GFP-Importinβ1 (Co-IP)
-130 KDa
-100 KDa
GFP-Importinβ1 (Lysate)
-130 KDa
-100 KDa
FLAG-TRAF6 (IP)
-70KDa
-55 KDa

Fig.7B

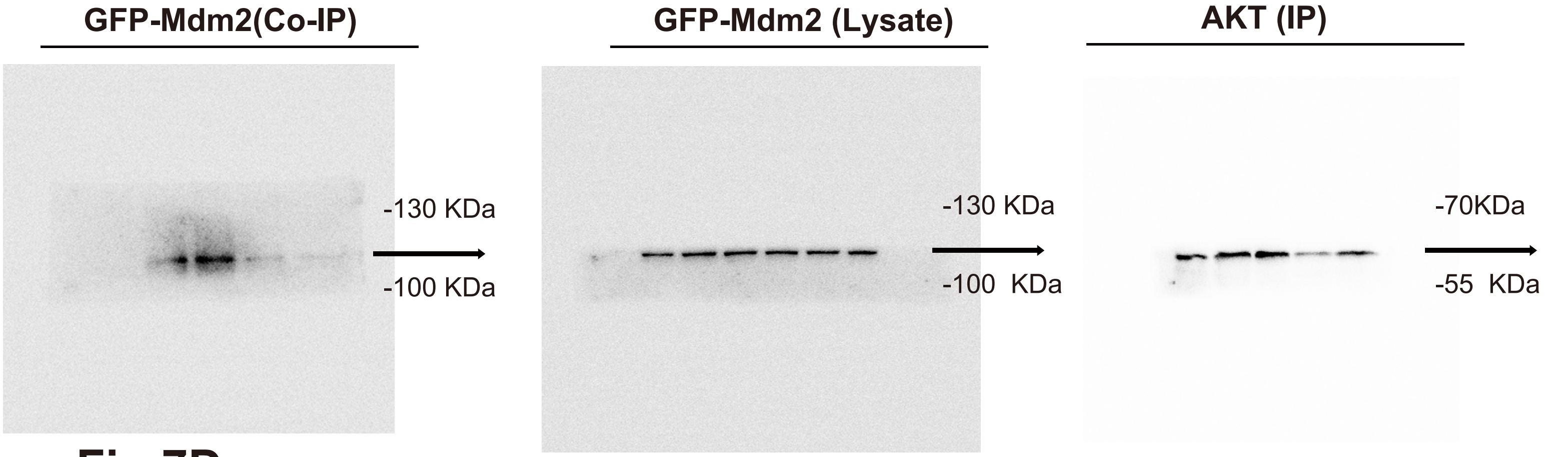

Fig.7D

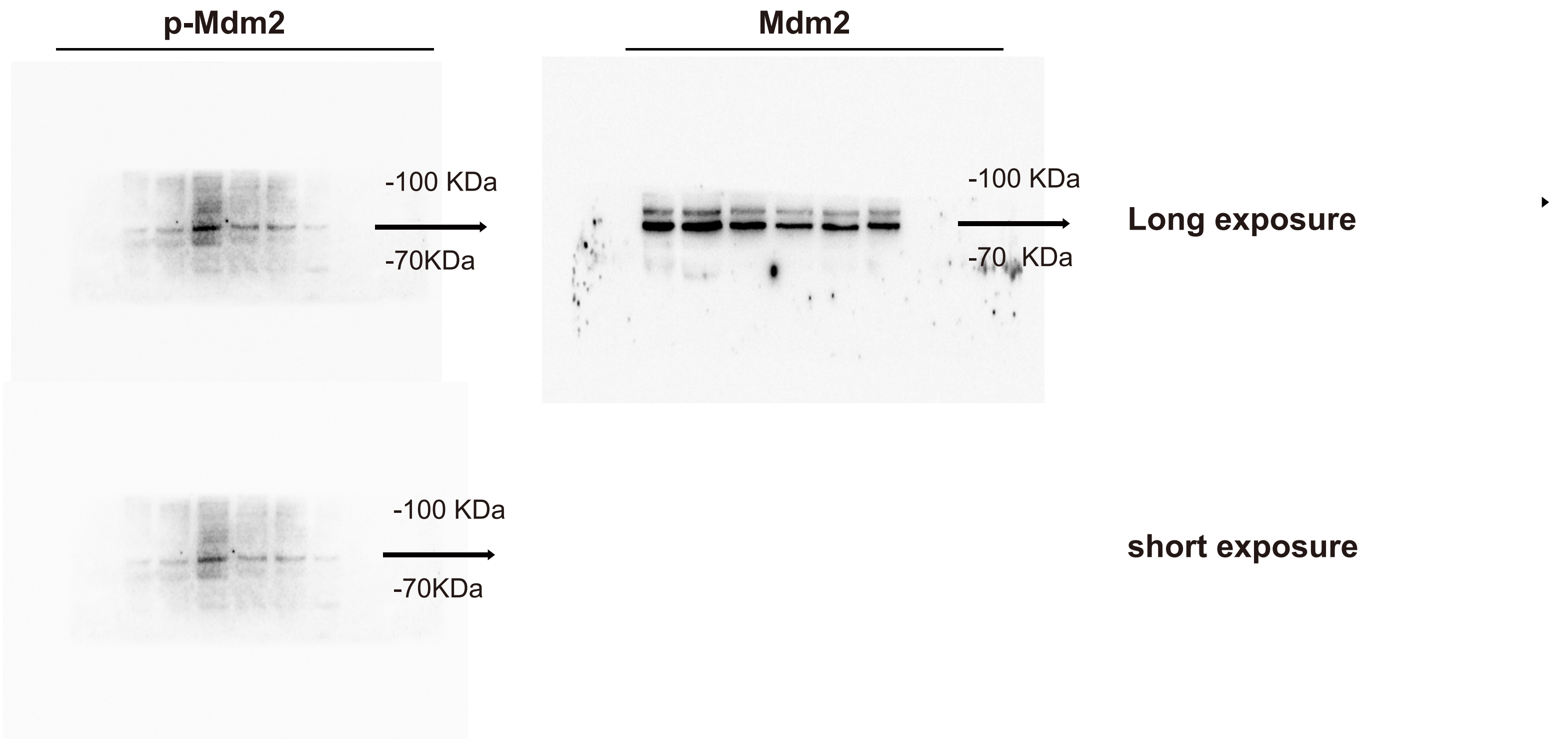

Fig.7E

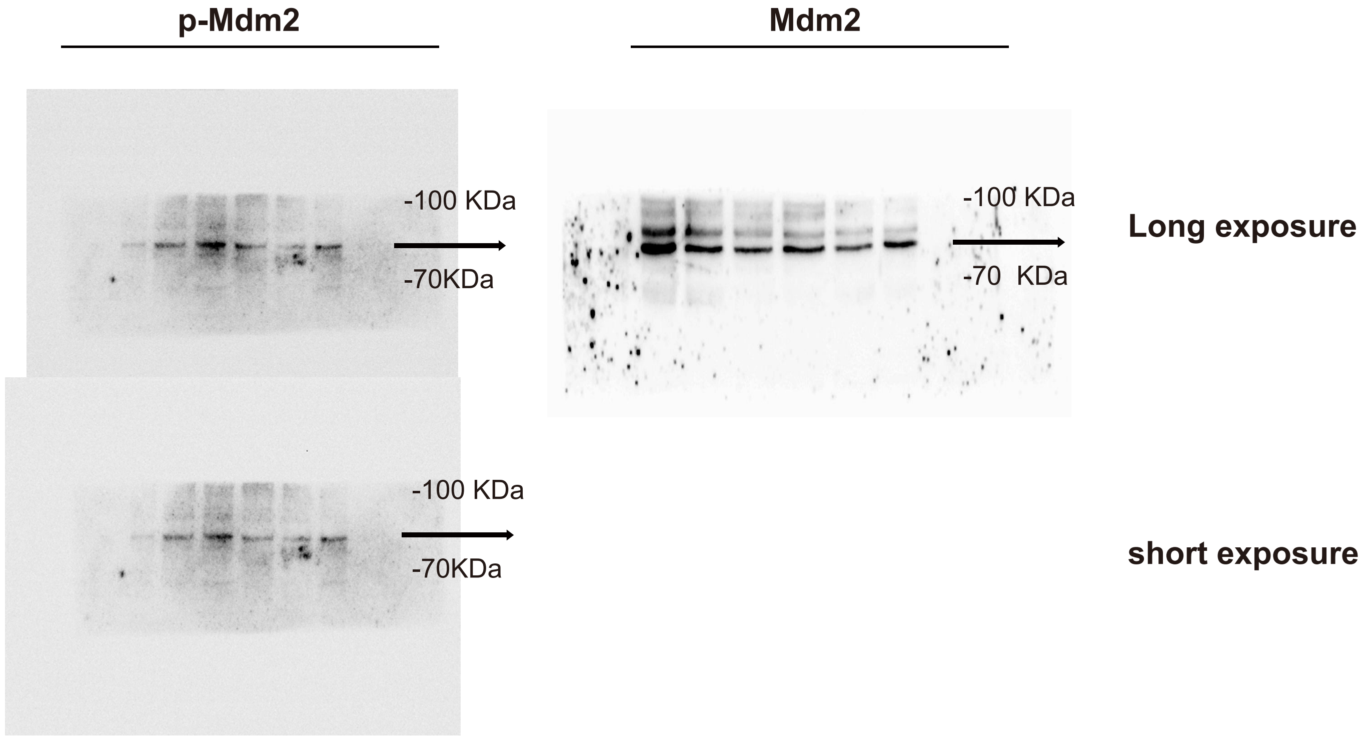

Fig.8B

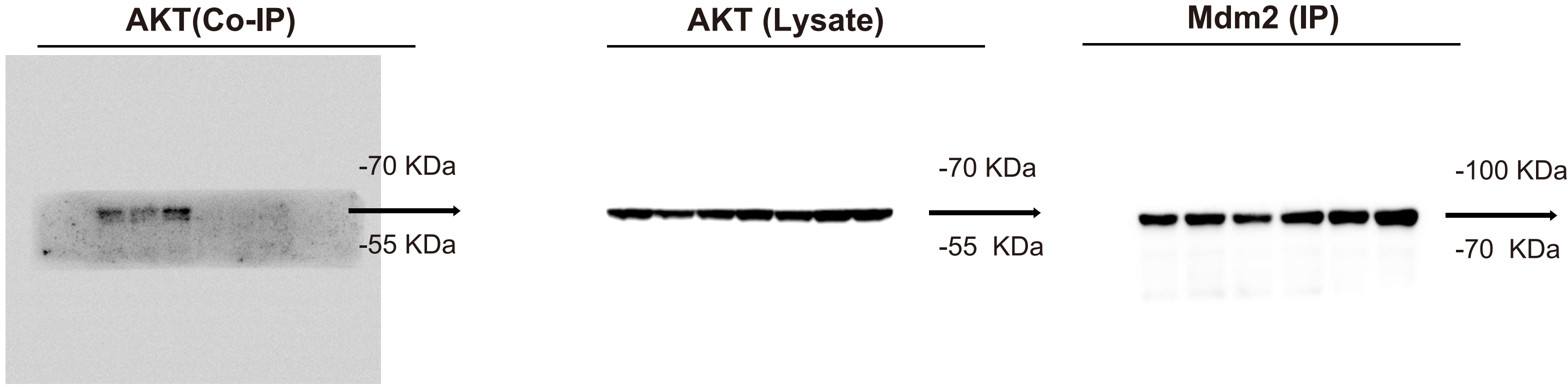

Fig.8C

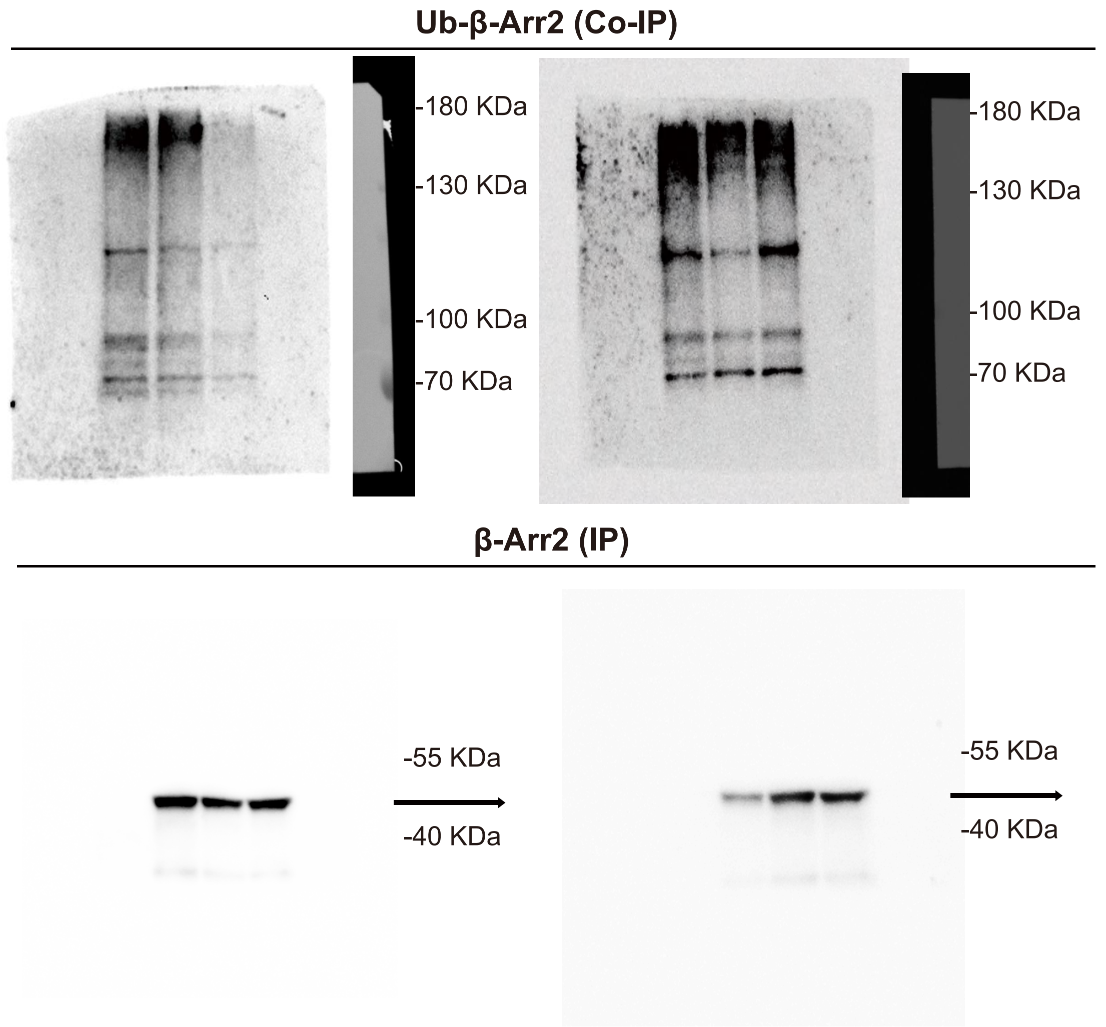

Fig.8D

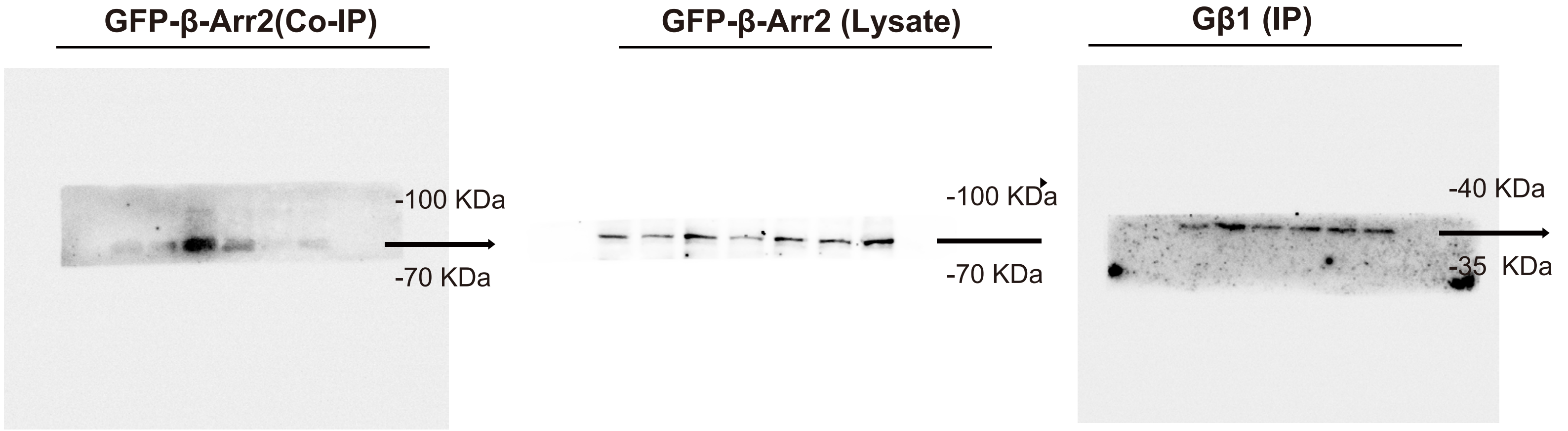

Fig.8E

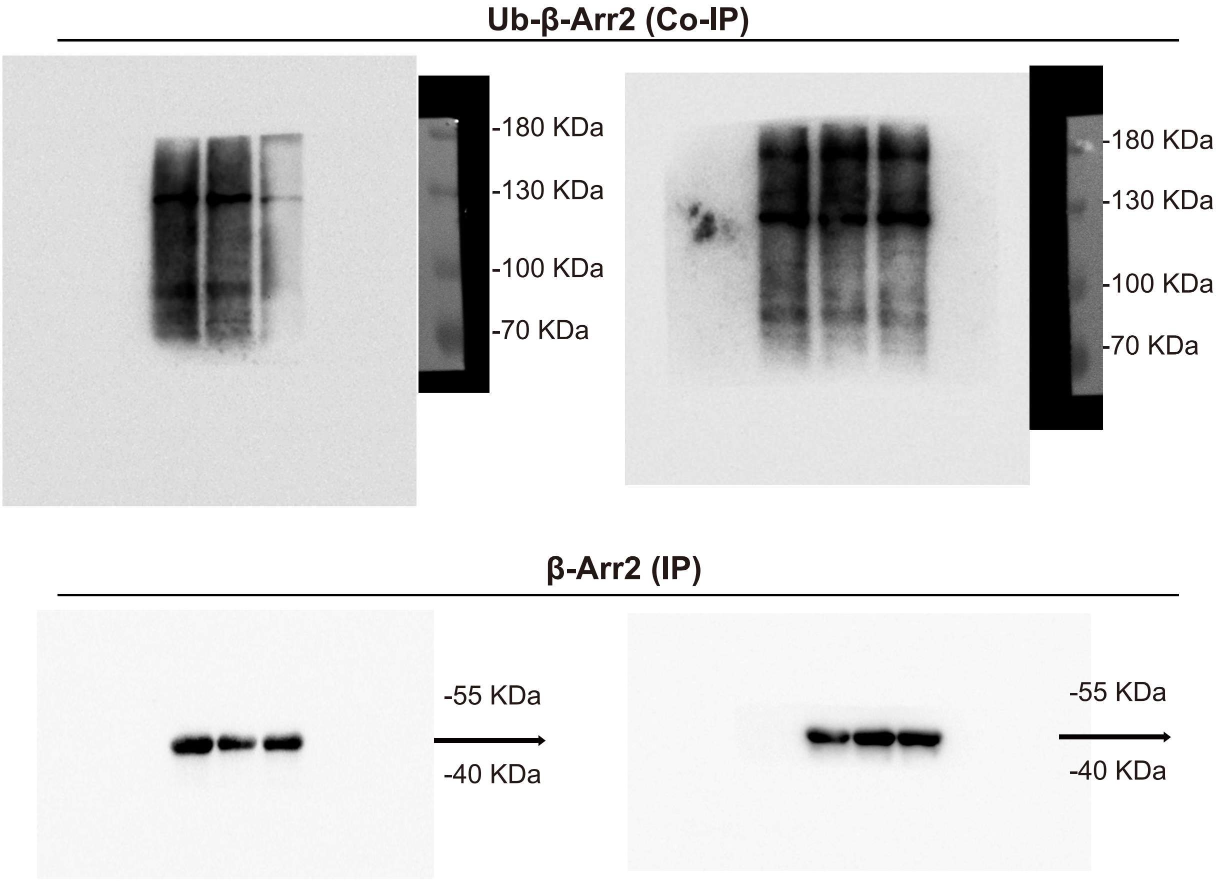
Ub-β-Arr2 (Co-IP)
-180 KDa
-130 KDa
-100 KDa
-70 KDa
-180 KDa
-130 KDa
-100 KDa
-70 KDa
β-Arr2 (IP)
-55 KDa
-40 KDa
-55 KDa
-40 KDa
